# Pirh2 modulates the mitochondrial function and cytochrome c-mediated neuronal death during Alzheimer’s disease

**DOI:** 10.1038/s41419-024-06662-1

**Published:** 2024-05-13

**Authors:** Abhishek Singh, Shubhangini Tiwari, Sarika Singh

**Affiliations:** 1 Division of Toxicology and Experimental Medicine, CSIR-Central Drug Research Institute, Lucknow, 226031 India; 2 Academy of Scientific & Innovative Research (AcSIR), Ghaziabad, 201002 India

**Keywords:** Cell death in the nervous system, Alzheimer's disease, Ubiquitylation

## Abstract

Pirh2 is an E3 ubiquitin ligase known to regulate the DNA damage responses through ubiquitylation of various participating signaling factors. DNA damage is a key pathological contributor to Alzheimer’s disease (AD), therefore, the role of Pirh2 was investigated in streptozotocin and oligomer Aβ_1–42_ induced rodent experimental model of AD. Pirh2 protein abundance increased during AD conditions, and transient silencing of Pirh2 inhibited the disease-specific pathological markers like level of p-Tau, βamyloid, acetylcholinesterase activity, and neuronal death. Biochemically, Pirh2 silencing significantly attenuated the oxidative stress, depleted mitochondrial membrane potential, cytochrome c translocation from mitochondria to cytosol, and depleted mitochondrial complex-I activity, and ATP level. Pirh2 silencing also inhibited the altered level of VDAC1, hsp75, hexokinase1, t-Bid, caspase-9, and altered level of apoptotic proteins (Bcl-2, Bax). MALDI-TOF/TOF, co-immunoprecipitation, and UbcH13-linked ubiquitylation assay confirmed the interaction of Pirh2 with cytochrome c and the role of Pirh2 in ubiquitylation of cytochrome c, along with Pirh2-dependent altered proteasome activity. Additionally, Pirh2 silencing further inhibited the translocation of mitochondrion-specific endonuclease G and apoptosis-inducing factors to the nucleus and DNA damage. In conclusion, findings suggested the significant implication of Pirh2 in disease pathogenesis, particularly through impaired mitochondrial function, including biochemical alterations, translocation of cytochrome c, endonuclease G and apoptosis-inducing factor, DNA damage, and neuronal apoptosis.

## Highlights

- Pirh2 is up-regulated in STZ and Aβ_1–42_ induced in vitro and in vivo experimental models of AD.
- Transient silencing of Pirh2 inhibited the pathological hallmarks like level of p-tau, β-amyloid, and acetylcholinesterase activity.
- Pirh2 silencing inhibited AD-related biochemical alterations, mitochondrial dysfunctions, and proteasome activity.
- Pirh2 upregulation is involved in cytochrome c translocation and its ubiquitylation and facilitates neuronal apoptosis.
- Pirh2 silencing also mitigates the DNA damage and inhibits the translocation of endonuclease G and AIF caused by STZ and Aβ_1–42_ in N2A cells.

## Introduction

Alzheimer’s disease (AD) is an age-related progressive neurologic disorder that causes the shrinkage and death of the cholinergic neuronal population associated with memory formation and retention [1–3]. The neuropathological hallmarks include the formation of amyloid plaques and hyperphosphorylation of microtubule-associated protein Tau [4, 5]. Disease pathology involves various biochemical alterations such as oxidative stress, defects in mitochondrial dynamics, the impaired ubiquitin-proteasome system (UPS), and the lysosomal pathway [6–8]. Both UPS and the lysosomal pathways are required to maintain the cellular as well as mitochondrial protein quality through the clearance of defective proteins, a mechanism termed autophagy and mitophagy, respectively, to sustain cellular homeostasis. In agreement, a recent report has shown that excessive ROS generation, energy crisis, altered calcium homeostasis, and mitochondrial dysfunction collectively aggravate AD pathogenesis and intensify cholinergic neuronal death [9].

Recent shreds of evidence reported the presence of ubiquitin residues in both plaques and tangles, suggesting the cohesion of ubiquitin and related modifications on the disease-linked signaling proteins, which conjointly participate in Tau hyperphosphorylation, amyloid aggregation, and consequent neuronal apoptosis [10]. The transfer of ubiquitin to signaling/substrate proteins is regulated by E3 ubiquitin ligases enzyme with the upstream enzymatic reactions of enzyme E1 and E2, the process called Ubiquitylation, an essentially required physiological mechanism of protein degradation [11, 12]. Previous findings have shown a close association between regulatory E3 ubiquitin ligases and AD pathology, particularly in the context of protein aggregation-dependent neuronal death [13]. However, the role of very few E3 ubiquitin ligases has been elucidated in AD pathology. It has been shown that the downregulation of E3 ubiquitin ligase E6-AP/Ube3A resulted in the loss of synaptic functions and cognitive defects in mouse models of AD [14]. E3 Ubiquitin ligases Parkin, Crl4^Crbn^ protein complex, Stub1, Nedd4, and Hrd1 also display significant interaction with AD-related neurodegenerative signaling proteins and leading to their altered expression implicating their role in disease pathogenesis [15, 16].

Pirh2 (p53-induced RING-H2 protein) is a RING domain-containing E3 ligase encoded by RCHY1 (RING-finger and CHY-zinc-finger domain-containing protein 1) gene in humans. Both N and C terminal domains of Pirh2 are responsible for protein-protein interaction, whereas the RING domain exhibits ubiquitin ligase activity [17]. Pirh2 ubiquitylates p53 (DNA damage repair protein) and targets it for proteasomal degradation [18]. It also degrades the active form of p53 during DNA damage and ubiquitylates the oncogenic isoform of p53. Besides p53, Pirh2 has also been shown to ubiquitylate members of the p53 protein family-like p63 and p73 and regulate their function [19–21]. Pirh2 has also been known to degrade several proteins, including Polη, Chk2, p27^kip1^, and HDAC1, that are reported to be involved in DNA damage responses, regulation of gene expression, cell cycle regulation, and tumor transformation [22–26] however, regarding AD the role of Pirh2 has not been investigated yet.

The role of Pirh2 in mitochondrial respiratory function through p53 has been suggested [27]. Though, the lineal role of Pirh2 with mitochondrion-associated apoptotic proteins has not been reported. However, p53-mediated alteration in mitochondrial physiology, like depletion of mitochondrial membrane potential and cytochrome c release mediated apoptosis has been reported during stressed conditions [28, 29]. Since AD pathology is well associated with energy crisis [30, 31], it is, therefore, suggested that p53 and its ubiquitylating partner Pirh2 may alter mitochondrial functionality during pathological conditions. Coherently in AD pathology, the augmented level of p53, as well as the impaired mitochondrial functions and DNA damage, have been observed [32–35] however, the role of Pirh2 is not yet investigated.

In context to mitochondrial functionality, previously, we and others have reported the cytosolic translocation of cytochrome c during AD pathology along with reduced mitochondrial membrane potential [36–39], which downstream may initiate the caspase-9 dependent neuronal apoptosis [37, 40]. Therefore, the present study was conducted to understand the mechanistic role of Pirh2 in AD pathology by employing both cellular and rodent experimental models. The primary objective of the study was to understand the specific role of Pirh2 on AD-specific pathological markers. Secondly, we have investigated the effect of Pirh2 on AD related biochemical alterations, mitochondrial functionality, its interacting proteins, apoptosis signaling, and DNA damage. Since depleted mitochondrial membrane potential causes the endonuclease G and apoptosis-inducing factor (AIF) mediated DNA damage, therefore, the effect of Pirh2 on these was also investigated to target mitochondrial-associated neuronal death during AD.

## Materials and methods

### Cell culture and treatments

The mouse neuroblastoma Neuro 2 A or SH-SY5Y cells were cells procured from the National Centre of Cell Science, Pune, India, and maintained at CDRI. Subsequent passages were done utilizing DMEM/F-12 culture media supplied with 10% FBS at 37 °C and 5% CO_2_. All the experiments were performed between passages 3–12 and the culture medium was changed on an alternative day to assert the healthy stipulation of cells. Streptozotocin (STZ) or oligomeric form of Aβ_1-42_ peptide treatment was given at a concentration of 1 mM and 100 nM for 48 and 24 hours, respectively, in N2A cells [39, 41, 42]. MG132 treatment was given in N2A cells at 5 mM concentration preceding 6 h from the respective experiment initiation to inhibit the endogenous proteasome activity. SH-SY5Y cells were treated with 1 mM concentration of STZ for 48 h and 100 nM concentration of Aβ_1–42_ for 24 h.

Oligomer Aβ_1–42_ Preparation: Aβ_1–42_ peptide were purchased from Sigma and initially dissolved to 1 mM in hexafluoroisopropanol and separated into aliquots in sterile microcentrifuge tubes, and hexafluoroisopropanol was removed under vacuum in a speed Vac and film was stored desiccated at 20 °C. For the Aβ_1–42_ oligomer preparation, each film was dissolved in F-12 (phenol red-free) to the final concentration of 100 µM and incubated at 4 °C for 24 h for treatment [41, 42]. For in vivo studies, peptide or film was dissolved in 0.1 M phosphate buffer to the final concentration of 200 µM incubated at 4 °C for 24 h, and then utilized for injection in rat brain [43].

List of chemicals and antibodies used for immunofluorescence and immunoblot analysis with their appropriate dilutions and observed molecular weight are mentioned in Supplementary table 1.

### siRNA mediated knockdown

Pre-designed siRNA constructs used for RNA interference were purchased from Invitrogen (Cat#: AM16708; Assay ID 102888) along with a negative control (Cat#: 4390843). Transient silencing of Pirh2 was done using Lipofectamine^TM^ RNAiMAX transfection reagent (Catalog no. 13778075).

#### Plasmids, cloning, and transfection

Myc-DDK-tagged wild-type mouse Pirh2 (Myc-DDK-Pirh2), plasmid constructs were purchased from OriGene technologies. The plasmids were propagated in DH5α cells, followed by their isolation using a commercial plasmid isolation kit (Promega, USA). For ectopic expression of the transients, the Myc-DDK-Pirh2 plasmids were transfected in N2A cells using lipofectamine 3000 based on manufacturer instructions. The total RNA was extracted after 24 h of transfection. The cDNA was obtained by RTPCR and amplified through PCR using respective primers (OriGene Technologies) followed by agarose gel electrophoresis to assess the gene expression. Protein level was estimated by western blotting in transfected N2A cells. An empty vector, PcDNA3.1 was used where necessary in order to adjust the DNA amounts.

### Mitochondrial dehydrogenase activity (MTT assay)

Cell viability was estimated using (3-(4,5Dimethylthiazol-2-yl)-2,5-Diphenyltetrazolium bromide) (MTT) dye [35]. Following treatment, the absorbance was read at 550 nm wavelength by a spectrophotometer (Eon, BioTek), and cell viability was analyzed using the observed mean of optical density.

### Acetylcholinesterase (AChE) activity estimation

Following transient transfection and treatment, the cells were rinsed with PBS, sonicated in 0.1 M sodium phosphate buffer containing 0.1% Triton-X-100, and centrifuged at 20,000 × rpm for 20 minutes at 4 and supernatant was collected and protein estimation was performed using Lowry method. Similarly, cortex and HP region of SD rats brain were isolated and homogenated in 0.1 M sodium phosphate buffer followed by centrifugation at 20,000 × rpm for 30 minutes at 4 °C and supernatant was collected and protein estimation was performed using Lowry method. Finally, the reaction mixture was prepared consisting of supernatant, 100 µM 5,5-dithio-bis-(2nitrobenzoic acid) (DTNB), and 0.1 M sodium phosphate buffer (pH 7.4) followed by incubation at 37 °C for 30 minutes in dark. Following incubation 20 mM acetylthiocholine iodide was added in the reaction, and enzymes kinetics was measured at 412 nm for 2 minutes at an interval of 15 seconds. The specific activity of AChE was calculated in µmoles/min/mg of protein [39, 44].

### Estimation of mitochondrial membrane potential

Mitochondrial membrane potential (Δ*ψ*_m_) was measured by fluorescent dye Rhodamine 123 and JC1. Following treatment, the Rhodamine 123 dye (10 µg/ml) was added to the cells and incubated in the dark for 1 h at 37 °C, followed by washing of cells with Krebs ringer (KR) buffer. The fluorescence intensity was measured using a fluorimeter at excitation and emission of 508 and 530 nm wavelength, respectively, and fluorescence images were captured by microscope (Nikon Eclipse E200) using a digital camera [35, 45]. For JC1 staining, after treatment, the cells were rinsed with PBS and incubated for 30 minutes in dye (1 µM) dissolved in PBS, followed by washing of the cells thrice, and the fluorescence intensity was measured at excitation and emission of 514 and 590 nm wavelengths, respectively, by fluorimeter for estimating JC1 aggregate. While for JC1 monomer the readings were taken at excitation and emission at 514 and 529 nm wavelengths, respectively. Fluorescence images were captured by microscope (Nikon, Eclipse E200).

### ATP assay

ATP assay was performed in scrambled and siRNA-Pirh2 transfected cells followed by STZ treatment for 48 h or Aβ_1–42_ treatment for 24 h using a commercially available kit (Biovision) following given instructions. Briefly, the cells were rinsed with PBS and lysed in ATP assay buffer followed by a deproteinization step using perchloric acid (PCA). Furthermore, excess PCA was removed by adding ice-cold 2 M KOH followed by centrifugation at 13,000 × *g* for 15 minutes at 4 °C, and the fluorimetric assay was performed [35]. Fluorescence intensity was estimated using fluorimeter (Varian Cary Eclipse, USA) at excitation 535 nm and emission 587 nm wavelengths.

### Mitochondrial complex-I activity

Mitochondrial complex-I activity was performed in isolated mitochondrial fractions from transfected N2A cells using mitochondrial isolation buffer (212 mM mannitol, 75 mM sucrose, 20 mm HEPES, 1 mM EDTA, 1 mM PMSF, and protease inhibitor cocktail 1 µl) [35]. The isolated mitochondria were further lysed in mitochondrial isolation buffer following five freeze-thaw cycles to disrupt the mitochondrial membranes, and protein concentration was measured using the Lowry method. The 200 µl reaction mixture was setup consisting of 35 mM phosphate buffer, 2.65 mM NaCN, 5 mM MgCl_2_, 1 mg/ml BSA, 0.4 µl of 1 mM antimycin A, 0.05 mM ubiquinone-1 and 10 µl of sample. The reaction mixture was incubated for 10 minutes at 37 °C in dark, and the reaction was initiated by the addition of 20 µl of 5 mM NADH. The kinetic profile of enzymatic activity was measured at 340 nm for 3 minutes at intervals of 15 seconds each by spectrophotometer (Eon, BioTek, USA).

### Reactive oxygen species (ROS)

Intracellular ROS level was measured by dichloroflluorescin diacetate (DCF-DA) dye in transiently knockdown N2A cells with scrambled and siRNA-Pirh2, followed by STZ or Aβ_1–42_ treatment for 48 h and 24 h, respectively. The cells were rinsed with Kreb’s ringer (KR) buffer and incubated in KR buffer with 50 µM of DCF-DA dye in a reaction volume of 200 µl for 2 h at 37 °C in the dark. The fluorescence intensity of the dye was measured by fluorimeter (Varian Cary Eclipse, USA) at excitation of 485 nm and emission at 520 nm wavelengths, respectively. DCF-DA fluorescence images were also captured by microscope (Nikon Eclipse E200). Mitochondrial ROS generation was assessed with MitoSOX^TM^ Red (Invitrogen, M36008), a fluorogenic dye for highly selective detection of mitochondrial superoxide. The cells transfected with scrambled and siRNA-Pirh2 followed by STZ or Aβ_1–42_ treatment were incubated with MitoSOX^TM^ Red (2 µM, 15 minutes, 37 °C) and then washed twice with PBS buffer and fluorescence intensity of dye was measured by fluorimeter (Varian Cary Eclipse, USA) at excitation of 510 nm and emission at 580 nm wavelengths, respectively [46].

### Glutathione (GSH) estimation

Briefly, after treatment, the cells were washed with PBS, collected in 0.1 M sodium phosphate buffer, and sonicated. The supernatant was mixed with 4% trichloroacetic acid in 1:1 proportion (v/v) and kept at 4 °C for 1 h. Following incubation, the cells were centrifuged at 4000 × *g* for 10 minutes at 4 °C and the reaction mixture was prepared by adding 10 µl supernatant, 1 mM DTNB, and 0.1 M phosphate buffer (pH 7.4) in the total reaction mixture of 200 µl. The absorbance was read at a wavelength of 412 nm utilizing an ELISA plate reader (Bio-Tek instruments). GSH concentration was extrapolated using a standard curve of known concentration of GSH standard, and results are expressed as GSH/µg/mg of protein [47].

### Lipid peroxidation (MDA)

Cell lysate was prepared in 100 µl of 1.15% KCl, and protein concentration was measured using Lowry method and remaining supernatant was mixed with phosphoric acid: TBA cocktail (3:1) and subsequently boiled at 100 °C in the water bath for 45 minutes. The samples were placed on ice for 10 minutes, and n-butanol (1:1) was added and centrifuged for 15 minutes at 4000 × rpm. The supernatant was placed in 96-well plate, and absorbance was read at 532 nm using a spectrophotometer (Econ, biotech, USA) [48].

### Western blotting

Following transient transfection with scrambled and siRNA-Pirh2 followed by STZ or Aβ_1–42_ treatment, the cells were scrapped, collected in culture medium, and centrifuged at 3000× rpm for 10 minutes at 4 °C. The pellet was rinsed with PBS lysed in lysis buffer, and then centrifuged at 15000 × *g* for 20 minutes at 4 °C. The cortex and HP region of the rat brain were isolated, homogenated in lysis buffer, and centrifuged for 20,000 × *g* for 30 minutes at 4 °C. The supernatant was taken and protein concentrations were determined by the Lowry method (1951). The Cells or tissue lysate, immunoprecipitants, or cellular fractions were loaded on SDS-PAGE, and the proteins were transferred to polyvinylidene difluoride (PVDF) membrane. PVDF was blocked with PBS-Tween containing 5% BSA for 1 h at room temperature (RT) and incubated with primary antibodies overnight at 4 °C. Further, the membrane was washed with PBS-T and incubated in respective horseradish peroxidase-conjugated secondary antibodies for 1 h at RT. The blots were visualized under ChemiDOC XRS+ (bio-red). The Mean intensity of bands was analyzed and normalized by β-actin using ImageJ software (NIH, USA).

Mitochondrial and cytosolic fractions were isolated according to protocol from Almeida and Medina 1998.

### Animals and stereotaxy

Male Sprague-Dawley rats weighing 200–220 g and aged 7–8 weeks were procured from the “National Laboratory Animal Centre” of CSIR—Central Drug Research Institute (CDRI). The experiments were conducted in accordance with ARRIVE guidelines, following international ethical standard protocols after approval from the CSIR-CDRI animal research ethics committee (IAEC/2018/89). Three animals were maintained in one polyacrylic cage with food and water ad libitum. Standard ambient conditions with 12 h light and dark cycle, room temperature 22 ± 1°C, and humidity 60–65% were provided. The rats were divided into two groups having minimum of 3 animals each: Control and STZ or Aβ_1–42_. The rats were anesthetized using a mixture of xylazine (10 mg/kg) and ketamine (80 mg/kg) and kept on the stereotaxic apparatus (Stoelting, USA), coordinates measured from the Bregma [49] and STZ (dissolved in ACSF 3 mg/kg) or Aβ_1–42_ (675 ng per rat) was administered in rat brain through intracerebroventricular route bilaterally [43, 50]. Appropriate care of rats was taken to prevent mortality, and after 21 days of STZ or 7 and 21 days of Aβ_1–42_ administration, the rats were anesthetized, followed by intra-cardiac perfusion with 0.9% saline and further decapitated to remove the brain. Cortex and HP (hippocampus) were isolated from rat brains, and the tissues were processed for various assays.

### Immunohistochemistry (IHC)

The sections were washed in PBS and fixed with 4% PFA in PBS for 20 minutes at room temperature. The brain sections were then permeabilized with PBS- 0.01% Triton X-100 for 5 minutes, incubated in blocking buffer (5% BSA in PBS- Triton-X-100) for 1 h, washed again with PBS-Triton-X-100 thrice for 5 minutes and incubated overnight at 4 °C with primary antibodies. Next day, the tissue sections were incubated with appropriate anti-mouse or anti-rabbit secondary antibodies for 1 h and washed with PBS-Triton-X-100 thrice for 5 minutes each. Anti-fade DAPI containing mounting medium was used to mount the slide, and the images were visualized and captured on a fluorescent microscope (Nikon Eclipse E200, Japan) [51].

### Immunofluorescence and confocal microscopy

The N2A cells were seeded on poly-l-lysine coated coverslips and transiently knockdown with scrambled and siRNA-Pirh2, followed by STZ treatment for 48 h and Aβ_1-42_ for 24 h. Then the cells were incubated in mitotracker deep red for 30 minutes at 37 °C. After that, the cells were rinsed with PBS and fixed with 4% PFA for 10 minutes at room temperature.

Cells were permeabilized with PBS-Triton-X-100 for 10 minutes blocked with 5% BSA for 1 h at room temperature, and incubated with respective primary antibodies overnight at 4 °C. Next day, the cells were washed with PBS-Triton-X-100 thrice for 5 minutes each and incubated with appropriate Alexafluor for 1 h at room temperature. After that, the cells were washed with PBS-Triton-X-100 and mounted on glass slides using an anti-fade mounting medium containing DAPI to stain the nuclei and analyzed through fluorescent microscopy (Nikon) using objective ×40 or confocal microscopy using objective ×100 (Leica) to visualize the fluorescence and co-localization of cytochrome c, AIF and endonuclease G with the mitochondrial marker mitotracker red [51].

### Comet assay

Following transfection with scrambled and/or siRNA-Pirh2 followed by STZ or Aβ_1–42_ treatment, the cells were washed with PBS. Cells (1 × 10^6^) were mixed with 200 µl of 0.8% low melting agarose kept at 37 °C, followed by layering of cells on the frosted side of frosted slides and kept at room temperature for 5 minutes with further incubation of 10 minutes on ice for solidification. The slides were then incubated in lysis buffer for 1 h and transferred to alkaline buffer for 20 minutes without current, followed by electrophoresis for 30 minutes using an alkaline buffer at 15 V and 25 mA. After electrophoresis, the slides were transferred in 0.4 M tris buffer (pH 7.5) for 10 minutes, and finally, the slides were kept in a humid chamber. Slides were stained with PI (40 µg/ml), and images were captured by microscope (Nikon Eclipse E200) using a digital camera olive tail moment was measured by comet score 2 software and analyzed by Graph Pad Prism 8 software [35, 45].

### Co-immunoprecipitation (Co-IP)

Following transfection with scrambled or siRNA-Pirh2 and treatment with STZ or Aβ_1–42_ in N2A cells, isolated brain tissue was lysed in lysis buffer, supplemented with phosphatases and proteases [35]. The extracts were centrifuged at 15,000 × *g* for 15 min at 4 °C. An equal amount of lysis extracts (2 mg) was incubated with anti-Pirh2, anti-cytochrome c, and anti-Flag antibody-protein G- sepharose beads complex with end-over-end rotation overnight at 4 °C. Bound proteins were washed with PBS 4–5 times, followed by 2 times washing with lysis buffer, for the removal of any non-specific binding. Then the immune complexes were boiled with 2× Laemmle buffer at 100 °C for 5 minutes to denature the proteins. The proteins were resolved on SDS-PAGE and immunoblotted with respective antibodies (cytochrome c, Pirh2) for visualizing the proteins using chemiluminescence substrate and observed by ChemDoc XRS + (Biorad) [51].

#### Mass spectrometry

##### Sample preparation

Samples resolved on SDS-PAGE were dipped in Coomassie blue (G-250) for 2 h followed by destaining (50:40:10: dH20: Methanol: Glacial acetic acid solution), after partial destaining the separated bands were sliced using a fine blade and immersed in microcentrifuge tubes again to remove the staining solution completely. Further, the gel slices were immersed in 100 µl of 25 mM ammonium bicarbonate (ABC) solution, followed by dehydrating the gels slices in 50 µl of solution A (2:1 mixture of acetonitrile: 50 mM ABC) for 5 minutes at RT. This step was repeated till the gel slices became transparent. Next, these gel slices were vortex for 5 minutes in 100% acetonitrile (ACN) and evaporated on a speed vacuum for 30 minutes at 45 °C to completely dehydrate them. These gel slices were rehydrated with 150 ng of trypsin and incubated on ice for 60 minutes. After the gel slices completely rehydrates, 50–100 µl of ABC solution was added and gel slices were incubated overnight at 37 °C. Following day, the supernatant was taken in a new centrifuge tube and dipped in 20–30 µl of 50% ACN and 2% tri-fluro acetic acid (TFA) and vortexed for 5 minutes. All these extractions were added to the centrifuge tube and evaporated on a speed vacuum till completely dry. The lyophilized samples were resuspended in 30% ACN and 0.1% TFA (5–6 µl) and again vortexed for 15 minutes to completely dissolve the sample. Sample (1 µl) was mixed with an equal amount of matrix {α-cyano-4hydroxycinnamic acid (α-CHCA), 1:1), spotted on a MALDI plate and processed using AB Sciex 4800 MALDI- TOF/TOF mass spectrometer. Spectra in positive mode over m/z 800–4000 Da were recorded.

From each spectrum, a maximum of 25 precursors with a minimum signal: noise ratio was carefully chosen for MS/MS analysis. Representative peptides spectrum against each sample was identified using protein and corresponding Pirh2 interacting proteins were predicted using MASCOT software [51].

##### Ubiquitylation assay

For ubiquitylation of cytochrome c, N2A cells and rat tissue samples were collected and lysed using lysis buffer. The extracts were centrifuged at 15,000 × *g* for 15 min at 4 °C. The total 3 mg of protein was taken for co-immunoprecipitation experiment and performed using anti-Pirh2 antibody employing protein G-Sepharose beads complex. The obtained immunocomplexes were rinsed with chilled PBS 4–5 times and then loaded on the vial containing the reaction components provided in the ubiquitylation assay kit (Enzo Life Sciences) based on the manufacturer’s instructions. The assay was performed at 37 °C for 60 minutes, and the reaction was terminated after the addition of 2× non-reducing laemmle buffer. The samples were resolved on SDS-PAGE, immunoblotted with ubiquitin antibody and also with cytochrome c antibody followed by their visualization using chemiluminescence [51].

##### Proteasome activity

Briefly, N2A cells were transiently knocked down with scrambled and siRNA-Pirh2, followed by STZ treatment for 48 h and Aβ_1–42_ for 24 h. Cells were scrapped, collected in the centrifuge tube, and centrifuged at 3000 × g for 5 minutes at 4 °C. The pellet was washed with PBS and lysed in lysis buffer, followed by centrifugation at 16,000 × *g* for 20 minutes at 4° C [52]. The supernatant was collected in a fresh centrifuge tube, and protein concentrations were determined using Lowry’s method (1951). Trypsin activity was estimated in lysis buffer supplemented with 2 mM ATP, 40 µg protein, and 100 µM of trypsin substrate (Z-ARR-AMC). Similarly, for chymotrypsin activity, 50 µM 7-amino, 4-methyl coumarin (SUC-LLVY-AMC) substrate was added in 50 μg of protein and lysis buffer supplemented with 2 mM ATP. The reaction mixture was incubated for 60 minutes at 37 °C in the dark, and the fluorescence intensity was measured using a fluorimeter (Varian Cary Eclipse, USA) at excitation and emission at 360/460 nm, respectively [52].

### Statistical analysis

Data were analyzed using Student’s unpaired *t* test or one-way analysis and two-way analysis of variance (ANOVA) as and when required, and the difference between control and treated sets was analyzed by post hoc Dunnett’s multiple comparisons or Newman Keul’s test or two-way ANOVA followed by Tukey’s multiple comparison test in experiments involving multiple treatments. Values are expressed as the mean ± SEM, and the *p* value <0.05 was considered statistically significant, and graphs were plotted using Graph Pad Prism 8 software.

## Results

### STZ and Aβ_1–42_ induced experimental model of AD and alteration in protein abundance of Pirh2

**Fig. 1:**
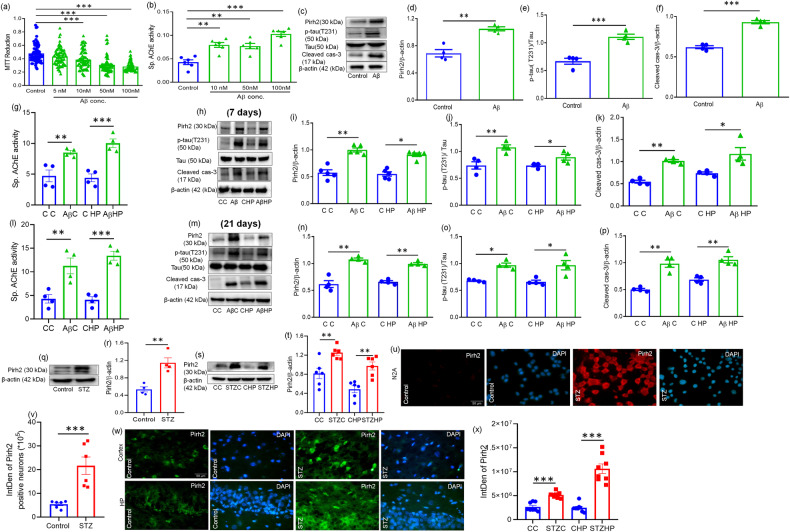
Pirh2 level is upregulated in N2A cells and rat brain during AD pathology. **a** Graphical representation for cell viability as estimated by mitochondrial dehydrogenase activity (MTT) after treatment with Aβ for 24 h in N2A cells, (Control *n* = 69; and Aβ (5 nM, 10 nM, 50 nM, and 100 nM) *n* = 69 from three independent experiments. Data were analyzed by one-way ANOVA followed by Dunnett’s post hoc test, ***p < 0.001 Control vs. Aβ. **b** Bar diagram showing the effect of Aβ in AChE activity in N2A cells, *n*_exp._ = 6. Data are represented as mean ± SEM and analyzed by one-way ANOVA followed by Dunnett’s post hoc test, **p < 0.01, ***p < 0.001 Control vs. Aβ. **c**–**f** Immunoblots & graph indicating the alternation in Pirh2, AD-specific pathological protein p-Tau, tau and neuronal apoptosis cleaved caspase-3 after treatment with Aβ for 24 h in N2A cells, *n*_exp._ = 4. Data are represented as mean ± SEM and analyzed by unpaired two-tailed student’s *t* test, **p < 0.01, ***p < 0.001 Control vs. Aβ. **g**, **l** Bar diagram showing the effect of Aβ in AChE activity in cortex and HP brain region after 7 & 21 days of Aβ administration respectively, Individual dots above the bar in 7 days and in 21 days are mean from four independent experiments *n*_exp._ = 4. Data are represented as mean ± SEM and analyzed by unpaired two-tailed student’s *t* test, **p < 0.01, ***p < 0.001 Control vs. Aβ. **h**–**k**, **m**–**p** Immunoblots & graph indicating the alternation in Pirh2, AD-specific pathological protein p-Tau, tau, and neuronal apoptosis cleaved caspase-3 in 7 days and 21 days of Aβ administration in rat brain respectively, *n*_exp._ = 4–5. Data are represented as mean ± SEM and analyzed by unpaired two-tailed student’s *t* test, *p < 0.05, **p < 0.01 Control vs. Aβ. Immunoblots and graphical illustration showing the level of Pirh2 in **q**, **r** N2A cells and **s**, **t** in rat brain cortex and HP region after treatment with STZ in comparison to control, normalized against β-actin; *n*_exp._ = 4–6, data are represented as mean ± SEM and analyzed by unpaired two-tailed student’s *t* test, **p < 0.01 Control vs. STZ. Immunofluorescent images (×40) graph depicting the expression of Pirh2 with counter staining of DAPI in **u**, **v** N2A cells and in **w**, **x** rat brain sections with or without STZ administration (Scale bar, 50 µm), Individual data point represents individual sampling in N2A cells Control *n* = 6; STZ *n* = 6 and in CC *n* = 8; STZC *n* = 8; CHP *n* = 8; STZHP *n* = 8 from three independent experiments (*n*_exp._ = 3). Quantifications are represented as mean ± SEM, and statistical analysis was performed using unpaired two-tailed student’s *t* test, ***p < 0.001 control vs. STZ. CC control cortex, STZ C streptozotocin Cortex, CHP control hippocampus, STZ HP streptozotocin hippocampus, STZ streptozotocin, Aβ Aβ(1–42)oligomer, AChE Acetylcholinesterase, p-tau (T231) phosphorylated-tau at threonine 231.

Neuronal cells [39, 41, 42] and rat experimental models of AD [43, 53] were employed, and AD pathology-specific parameters like protein level of β-amyloid and phosphorylated-Tau (pT-231) and acetylcholinesterase (AChE) activity were estimated. We performed MTT reduction assay for a dose selection of STZ and Aβ_1–42_ in N2A cells at 48 h and 24 h time points, respectively. A significant decrease in cell viability was observed at 0.01 mM, 0.1 mM, and 1 mM concentrations of STZ and at 50 nM and 100 nM concentrations of Aβ_1–42_. (Fig. S1a, Fig. 1a). However, a more significant increase in AChE activity was found at 1 mM STZ concentration therefore, all in vitro experiments were conducted at 1 mM concentration of STZ (Fig. S1b). Similarly, in Aβ_1–42_ treated N2A cells more significant increase in AChE activity was observed at 100 nM concentration of Aβ_1–42_ therefore, it was selected for further experiment in N2A cells (Fig. 1b). The protein abundance of phosphorylated-tau T-231, β-amyloid and amyloid precursor protein was increased in STZ-treated cells (Fig. S1c–f) and p-tau at T231 protein level was increased significantly in Aβ_1–42_ treated N2A cells, confirming the induction of AD pathogenesis in neuronal cells (Fig. 1c, e). The protein level of cleaved caspase 3 was also increased in STZ or Aβ_1–42_ treated N2A cells (Fig. 1Sc, g; Fig. 1c, f). For in vivo studies, AD pathology-specific parameters like protein level of amyloid precursor protein, β-amyloid and phosphorylated-Tau at T-231 and AChE activity were estimated, and all the parameters were increased in STZ-induced AD model (Fig. S2a–e). Similarly, AChE activity and phosphorylated-Tau at T-231 were increased in cortex and HP region after Aβ1–42 post injection of 7 days (Fig. 1g, h, j) and 21 days (Fig. 1l, m, o) post injection of Aβ_1–42_ in rat brain. Moreover, neuronal terminal apoptotic marker cleaved caspase 3 was also measured, and data showed significant neuronal death at 21 days of STZ, and at 7 days of Aβ_1–42_ and 21 days of Aβ_1–42_ post injection in rat brain (Fig. S2b, f; Fig. 1h, k, m, p).

After validation of STZ and Aβ_1–42_ induced Alzheimer’s disease model, we then checked the level of Pirh2 through immunoblotting in both N2A neuroblastoma cells and Sprague-Dawley (SD) rat brain regions (cortex and hippocampus) in both control and STZ or Aβ_1–42_ induced AD model. Data showed that Pirh2 was upregulated in Aβ_1–42_ and STZ-treated N2A cells (Fig. 1c, d, q, r). In parallel, the significantly upregulated protein level of Pirh2 was observed in both hippocampal and cortex regions of the rat model after 21 days of STZ and 7 and 21 days of Aβ_1–42_ administration (Fig. 1h, i, m, n, s, t). Immunofluorescence images collated with the observation of immunoblots suggesting the upregulated Pirh2 level in neuronal cells as well as in cortex and hippocampal regions of rat brain (Fig. 1u–x).

Additionally, protein abundance of Pirh2 was also increased in SH-SY5Y cells treated with STZ or Aβ_1-42_ (Fig. S3a–d). Data suggested that after STZ or Aβ_1–42_ treatment protein abundance of Pirh2 was significantly increased along with AD-specific pathological markers.

### Transient silencing of Pirh2 attenuates the AD-specific pathological markers

**Fig. 2:**
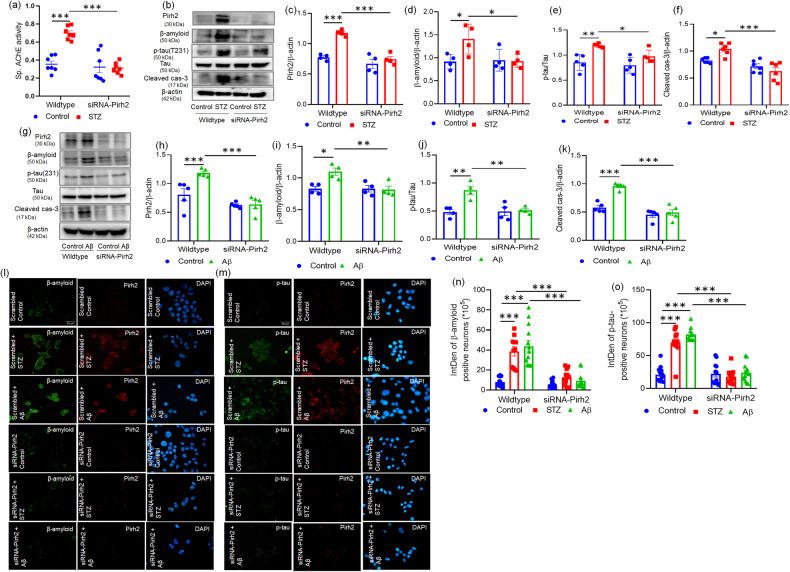
Effect of Pirh2 silencing on AD-specific pathological markers. **a** Graph showing the specific AChE activity in N2A cells after transient transfection with scrambled and siRNA-Pirh2 with or without STZ treatment, (scrambled control *n* = 7, scrambled STZ *n* = 7; siRNA-Pirh2 control *n* = 7; siRNA-Pirh2 STZ *n* = 7 from three independent experiments. **b**–**k** Immunoblots and graphical representations illustrating the protein level of Pirh2, AD markers (β-amyloid and p-tau), and neuronal death (cleaved caspase 3) in N2A cells after transient transfection with scrambled and siRNA-Pirh2 in N2A cells with or without STZ or Aβ treatment, *n*_exp_ = 4–6. **l**, **m** Immunofluorescent images (×40) depicting the expression of β-amyloid (green) and p-tau (T231) (green) and Pirh2 (red) in scrambled and siRNA-Pirh2 with or without STZ or Aβ treatment in N2A cells with counterstaining of DAPI (blue), *n*_exp._=3 (Scale bar, 50 µm). **n**, **o** Graph showing the quantifications of integrated density of β-amyloid (scrambled control *n* = 14; scrambled STZ *n* = 11; scrambled Aβ *n* = 13; siRNA-Pirh2 control *n* = 13; siRNA-Pirh2 STZ *n* = 12; siRNA-Pirh2 Aβ *n* = 12 are individuals sample from three independent experiments) and p-tau(T231) (scrambled control *n* = 14; scrambled STZ *n* = 11; scrambled Aβ *n* = 11; siRNA-Pirh2 control *n* = 12; siRNA-Pirh2 STZ *n* = 14; siRNA-Pirh2 Aβ *n* = 14 are individuals sample from three independent experiments) in neuronal N2A cells after transient transfection with scrambled and siRNA-Pirh2 with or without STZ or Aβ treatment. Quantifications of data are represented as mean ± SEM, and statistical analysis was performed using two-way ANOVA followed by Tukey’s test. *p < 0.05, **p < 0.01, ***p < 0.001 scrambled control vs. Scrambled (STZ or Aβ); scrambled (STZ or Aβ) vs. siRNA-Pirh2 (STZ or Aβ). STZ streptozotocin, Aβ Aβ(1–42) oligomer, AChE acetylcholinesterase, p-tau(T231) phosphorylated-tau at threonine 231, scrambled control siRNA.

Since, we observed the increased expression of Pirh2 with AD-specific pathological markers; further experiments were conducted to verify the precise role of Pirh2 in AD pathology. Therefore, the neuronal cells were transiently knockdown using siRNA targeting Pirh2 gene followed by STZ or Aβ_1–42_ treatment. STZ /or Aβ_1–42_ treatment caused significantly upregulated AChE activity and protein level of β-amyloid and p-Tau (T-231) in comparison to respective control cells. While contrary to this, the transient silencing of Pirh2 with STZ treatment inhibited the increased AChE activity in comparison to scrambled STZ-treated N2A cells (Fig. 2a). Similarly, transient silencing of Pirh2 followed by STZ or Aβ_1–42_ treatment in N2A cells inhibited the increased protein abundance of β-amyloid and p-Tau (T-231) compared to scrambled STZ or Aβ_1–42_ treated N2A cells (Fig. 2b–e, g–j). AD is also characterized by irreversible neuronal cell death, therefore, checked the level of cleaved caspase-3 in both scrambled and siRNA-Pirh2 groups with or without STZ or Aβ_1–42_ treatment. Transient knockdown of Pirh2 inhibited the STZ or Aβ_1–42_ induced upregulated level of cleaved-caspase 3 in comparison to scrambled STZ or Aβ_1–42_ treated cells (Fig. 2b, f, g, k). In addition, immunofluorescence images and graphs of β-amyloid and p-Tau (T-231) were also in affirmation of our aforementioned findings (Fig. 2l–o). The observed data demonstrate the specific role of Pirh2 in regulating the AD-specific pathological markers and therefore, the study was further done for mechanistic investigation.

### Pirh2 silencing inhibits the disease pathology related mitochondrial dysfunction and energy crisis

**Fig. 3:**
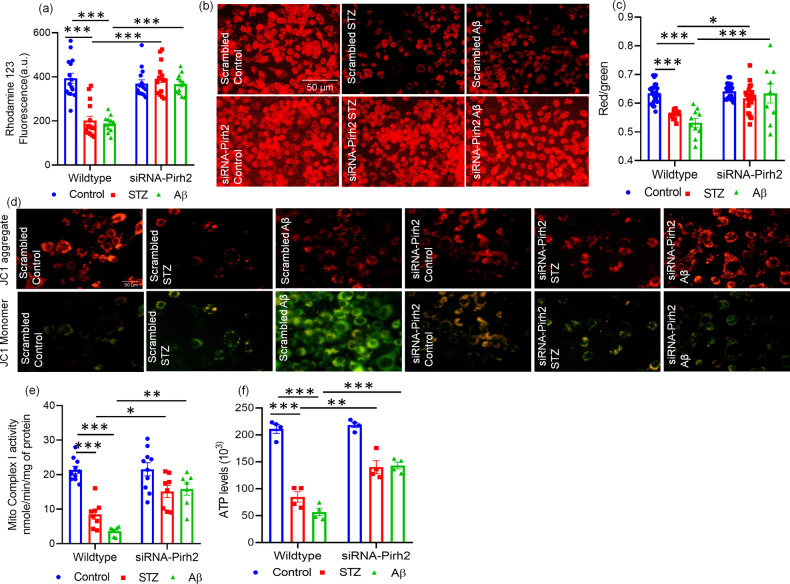
Effect of Pirh2 on mitochondrial physiology. **a**, **b** Bar diagram and fluorescent images (×20) represents ψ_m_ level (fluorescence of Rhodamine 123 dye) in scrambled and siRNA-Pirh2 transfected N2A cells representing the alteration in mitochondrial membrane potential after STZ or Aβ exposure in comparison to the respective control, *n*_exp_ = 3 (Scale bar, 50 µm), (scrambled control *n* = 15; control +siRNA-Pirh2 *n* = 15; scrambled STZ or STZ +siRNA-Pirh2 *n* = 15; scrambled Aβ or Aβ+siRNA-Pirh2 *n* = 12 from three independent experiments, *n*_exp._ = 3). **c** Graphical illustration (ratio of red and green fluorescence of JC1) in individuals replicates from scrambled control *n* = 26; scrambled STZ *n* = 11; scrambled Aβ *n* = 10; siRNA-Pirh2 control *n* = 28; siRNA-Pirh2 STZ *n* = 24; siRNA-Pirh2 *n* = 9 from three independent experiments and **d** fluorescent images (×40) of JC1 dye depicting the ψ_m_ level in scrambled and siRNA-Pirh2 with or without STZ or Aβ treatment, *n*_exp._ = 3 (Scale bar, 50 µm). **e** Bar diagram represents the mitochondrial complex-I activity in STZ or Aβ treated N2A cells, (scrambled control *n* = 10; control +siRNA-Pirh2 *n* = 10; scrambled STZ or STZ +siRNA-Pirh2 *n* = 8; scrambled Aβ or Aβ+siRNA-Pirh2 *n* = 7 from three independent experiments, *n*_exp._ = 3) and **f** ATP level after transient transfection with scrambled and siRNA-Pirh2 indicating STZ or Aβ induced energy depletion in comparison to the control N2A cells, *n*_exp_ = 4. All the data are represented as mean ± SEM, and statistical analysis were performed using two-way ANOVA, followed by Tukey’s test. *p < 0.05, **p < 0.01, ***p < 0.001 scrambled control vs. scrambled (STZ or Aβ) and scrambled (STZ or Aβ) vs. siRNA-Pirh2 (STZ or Aβ) STZ streptozotocin, Aβ Aβ(1–42) oligomer, ψ_m_ mitochondrial membrane potential, scrambled control siRNA.

The integrity of mitochondrial protein quality is widely regulated by UPS and the functions of both get impaired during neurodegenerative conditions [7, 54]. The interaction of Pirh2 and p53 in regulating mitochondrial machinery has also been reported [28, 29, 34]. Previously, we have reported significant mitochondrial dysfunction during AD pathogenesis in both cellular and rodent models [35]. Therefore, further experiments were conducted to assess the role of Pirh2 in mitochondrial health. STZ or Aβ_1–42_ treated N2A cells exhibited the depleted Δ*ψ*_m_, which was inhibited in siRNA-Pirh2 transfected neuronal cells with STZ or Aβ_1–42_ treatment (Fig. 3a–d). Fluorescent imaging of cells also collates with quantitative observations (Fig. 3b, d). Fluorescence images showing that a high ratio of red (aggregate)/green (monomer) stain of JC1 in the siRNA-Pirh2 treatment cells was an indicator of high Δ*ψ*_m_ as opposed to STZ or Aβ_1–42_ treated cells with high green/red ratio under reduced Δ*ψ*_m_ state (Fig. 3c, d). Further, to assess the mitochondrial function in terms of energy biogenesis, the mitochondrial complex-I activity was estimated. In agreement, a recent study also showed that impaired mitochondrial complex-I activity is largely associated with amyloid load and Tau deposition [55]. Observations showed that Pirh2 silencing in N2A cells significantly inhibited the decrease in mitochondrial complex-I activity caused by STZ or Aβ_1–42_ treatment (Fig. 3e). Next, we estimated the ATP level in neuronal cells as cholinergic neurons demand a high energy supply for cognitive function and mito-energetic failure during diseased condition accentuate the disease pathology and promotes neuronal death [35]. STZ or Aβ_1–42_ treatment in N2A cells accelerated the ATP depletion, which was de-escalated in the siRNA-Pirh2 transfected cells, followed by STZ or Aβ_1–42_ treatment (Fig. 3f).

Taken together, our data highlights the intricate participation of Pirh2 in modulating the mitochondrial function and energy metabolism defects in STZ and Aβ_1–42_-treated N2A cells.

### Pirh2 silencing attenuates the STZ and Aβ_1–42_ induced oxidative stress and cytochrome c translocation

**Fig. 4:**
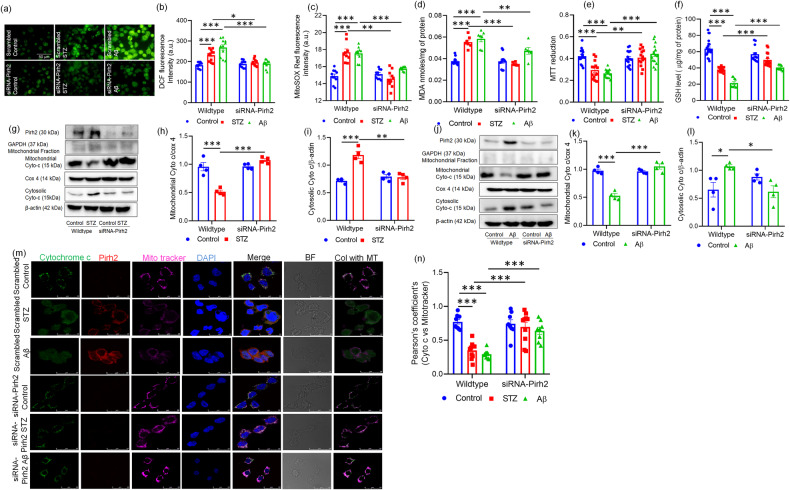
Effect of Pirh2 on oxidative stress and cytochrome c translocation. **a**, **b** Fluorescent images (DCF-DA) (×40) and graph depicting the ROS level in scrambled and siRNA-Pirh2 transfected N2A cells with or without STZ or Aβ treatment indicating the effect of Pirh2 silencing on STZ and Aβ induced oxidative stress, (scrambled control *n* = 11; scrambled STZ *n* = 11; scrambled Aβ *n* = 11; siRNA-Pirh2 control *n* = 10; siRNA-Pirh2 STZ *n* = 10; siRNA-Pirh2 Aβ *n* = 10 from three independent experiments, *n*_exp_ = 3 (Scale bar, 50 µm). **c** Graph showing the mitochondrial ROS level (MitoSOX red fluorescence) in scrambled and siRNA-Pirh2 transfected N2A cells with or without STZ or Aβ treatment, (scrambled control *n* = 10; scrambled STZ *n* = 9; scrambled Aβ *n* = 10; siRNA-Pirh2 control *n* = 9; siRNA-Pirh2 STZ *n* = 10; siRNA-Pirh2 Aβ n = 8 from three independent experiments, *n*_exp_ = 3. Bar diagram represents **d** Lipid Peroxidation (MDA) level in scrambled and siRNA-Pirh2 transfected N2A cells with or without STZ or Aβ treatment, (scrambled control *n* = 14; scrambled STZ *n* = 7; scrambled Aβ *n* = 7; siRNA-Pirh2 control *n* = 12; siRNA-Pirh2 STZ *n* = 6; siRNA-Pirh2 Aβ *n* = 6 from three independent experiments, *n*_exp_ = 3). Bar diagram represents **e** mitochondrial succinate dehydrogenase activity (MTT reduction) in scrambled and siRNA-Pirh2 transfected N2A cells with or without STZ or Aβ treatment, (scrambled control *n* = 15; scrambled STZ *n* = 14; scrambled Aβ *n* = 15; siRNA-Pirh2 control *n* = 15; siRNA-Pirh2 STZ *n* = 14; siRNA-Pirh2 Aβ n = 15 from three independent experiments, *n*_exp_ = 3. Bar graph represents **f** GSH level in scrambled and siRNA-Pirh2 transfected N2A cells with or without STZ or Aβ treatment, (scrambled control *n* = 24; scrambled STZ *n* = 15; scrambled Aβ *n* = 10; siRNA-Pirh2 control *n* = 15; siRNA-Pirh2 STZ n = 14; siRNA-Pirh2 Aβ *n* = 15 from three independent experiments, *n*_exp_ = 3. **g**, **j** Blots showing the level of Pirh2 in whole cell lysate and mitochondrial cytochrome c and cytosolic cytochrome c in cellular fractions and GAPDH blot showing the purity of mitochondrial fraction in respective set of samples and **h**, **i**, **k**, **l** bar diagram represents the quantification of mitochondrial cytochrome c and cytosolic cytochrome c protein abundance with respect to loading control cox 4 and β-actin respectively in scrambled and siRNA-Pirh2 with or without STZ or Aβ treatment in N2A cells, *n*_exp_ = 4. **m** Confocal microscopy images represent the translocation of cytochrome c from the mitochondria to the cytosol along with Pirh2 double-immunofluorescence in scrambled and siRNA-Pirh2 transfected N2A cells in the presence of mitotracker deep red and DAPI counterstaining after STZ or Aβ treatment compared to the control, *n*_exp_ = 3 (Scale bar, 25 µm). **n** Graph showing the Pearson correlation coefficient (PCC) between cytochrome c and mitotracker deep red in wildtype and siRNA-Pirh2 transfected N2A cell with or without STZ or Aβ treatment, (scrambled control *n* = 10; scrambled STZ *n* = 10; scrambled Aβ *n* = 10; siRNA-Pirh2 control *n* = 8; siRNA-Pirh2 STZ *n* = 10; siRNA-Pirh2 Aβ *n* = 8 from three independent experiments *n*_exp_ = 3). All the data are represented as mean ± SEM, and *P* values are calculated with statistical test two-way ANOVA followed by Tukey’s test. *p < 0.05, **p < 0.01, ***p < 0.001 scrambled control vs. scrambled (STZ or Aβ); scrambled (STZ or Aβ) vs. siRNA-Pirh2 (STZ or Aβ). STZ streptozotocin, Aβ Aβ(1–42) oligomer, Cyto c Cytochrome c, MDA Malondialdehyde, ROS Reactive oxygen species, GSH Glutathione, scrambled control siRNA, MT mitotracker deep red, Col colocalised.

Mitochondrial complex-I and complex-III are the major components of the electron transport chain (ETC) and largely cause ROS generation during oxidative phosphorylation, which is neutralized with cellular antioxidants in physiological conditions, majorly by glutathione (GSH). Previously we have reported the augmented ROS level in STZ induced AD model [35] therefore, the effect of Pirh2 on ROS generation was assessed. In agreement with our previous findings, we observed an increased cellular ROS generation in STZ or Aβ_1–42_ treated neuronal cells as estimated through fluorescent imaging and quantitative recordings (Fig. 4a, b). Contrary to this, siRNA-Pirh2 transfected cells exhibited the depletion in STZ or Aβ_1–42_ induced increased ROS generation. Mitochondrial ROS was also assessed in scrambled and siRNA-Pirh2 transfected N2A cells with or without STZ or Aβ_1–42_ treatment. Significantly increased mitochondrial ROS level was observed in STZ or Aβ_1–42_ treated N2A cells compared to control neuronal cells. However, in siRNA-Pirh2 transfected cells with STZ or Aβ_1–42_ treatment, significant decrease in mitochondrial ROS level was observed (Fig. 4c). Excessive ROS generation leads to lipid peroxidation. Therefore, we also checked the lipid peroxidation (MDA level) in scrambled and siRNA-Pirh2 transfected N2A cells. In STZ or Aβ_1–42_ treated cells, the MDA level was higher compared to control cells, while Pirh2 silenced cells exhibited a significantly decreased MDA level (Fig. 4d). Next, we checked the NADH-dependent succinate dehydrogenase activity in N2A cells to estimate the mitochondrial metabolic rate through MTT assay in both wild-type and Pirh2 silenced neuronal cells. STZ or Aβ_1–42_ treated cells exhibited a significant reduction in cell viability in comparison to the control N2A cells, which was counterbalanced in the siRNA-Pirh2 group with or without STZ or Aβ_1–42_ treatment (Fig. 4e). GSH homeostasis is primarily affected by oxidative stress therefore; further the effect of Pirh2 on GSH level was estimated. Findings showed that silencing of Pirh2 significantly attenuated the depleted GSH level in STZ or Aβ_1–42_-treated cells (Fig. 4f).

Since, previously we have observed the cytosolic translocation of cytochrome c from mitochondrion [39]. Here, in this study, we have investigated the effect of Pirh2 on the translocation of cytochrome c from mitochondria to cytosol. Therefore, first, we divided the total cell in two parts one for measuring the Pirh2 protein abundance in whole cell lysate and the second part for isolation of mitochondrial and cytosolic fraction. Immunoblot showing the depleted Pirh2 protein level in cell transfected with siRNA-Pirh2 followed by STZ or Aβ_1–42_ treatment (Fig. 4g, j). In the remaining cells, mitochondrial and cytosolic fractions were prepared in both wild-type and siRNA-Pirh2 transfected cell with or without STZ or Aβ_1–42_ treatment. The GAPDH blot showing the purity of mitochondrial fraction in different treated sets (Fig. 4g, j). We observed that STZ or Aβ_1–42_ caused significant translocation of cytochrome c from mitochondria into the cytosol, which was inhibited in Pirh2 silenced neuronal cells as observed by immunoblotting as well as by confocal microscopy (Fig. 4g–m). Pearson’s correlation coefficient for cytochrome c vs mitotracker deep red suggests that cytochrome c was significantly translocated from mitochondria in STZ or Aβ_1–42_ treated N2A cells compared to control N2A cells. However, in siRNA-Pirh2 transfected N2A cells with or without STZ or Aβ_1–42_ treatment cytochrome c was more colocalized with mitochondria (Fig. 4n). Data suggested that Pirh2 promote the translocation of cytochrome c in STZ or Aβ1–42 treated neuronal cells.

**Fig. 5:**
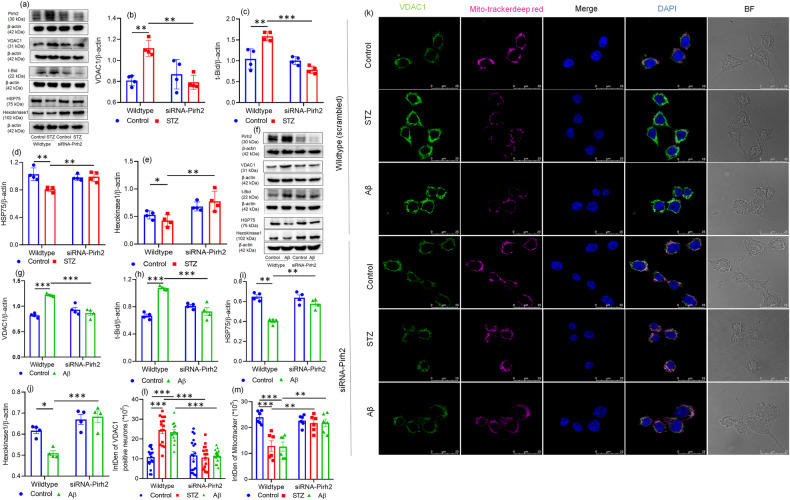
Pirh2 silencing altered the mitochondrial physiology-related protein. **a**–**j** Immunoblots and graphical representations illustrating the protein level of Pirh2, VDAC1, t-bid, HSP75, and Hexokinase1 in transiently transfected with scrambled and siRNA-Pirh2 in N2A cells with or without STZ or Aβ treatment, *n*_exp._ = 4. **k** Confocal images (×100) depicting the expression of VDAC1 (green), mitotracker deep red (magenta) with counterstain of DAPI (blue) and **l** integrated density of the VDAC1 neurons represent the expression of VDAC1 with or without STZ or Aβ treatment in scrambled or siRNA-Pirh2 transfected N2A cells; (scrambled control *n* = 19; scrambled STZ *n* = 15; scrambled Aβ *n* = 14; siRNA-Pirh2 control *n* = 18; siRNA-Pirh2 STZ *n* = 17; siRNA-Pirh2 Aβ *n* = 16, from three independent experiments) (scale bar 25 µm). **m** Integrated density of the mitotracker deep red represents the level of MMP with or without STZ or Aβ treatment in scrambled or siRNA-Pirh2 transfected N2A cells; *n* = 6 from three independent experiments. All the data are represented as mean ± SEM, and *P* values were calculated with statistical test two-way ANOVA followed by Tukey’s multiple comparision test. *p < 0.05, **p < 0.01, ***p < 0.001 scrambled control vs. scrambled (STZ or Aβ) and scrambled (STZ or Aβ) vs. siRNA-Pirh2 (STZ or Aβ). STZ streptozotocin, Aβ Aβ(1–42) oligomer, scrambled control siRNA.

Mitochondrial functioning is largely regulated by various mitochondrial proteins such as VDAC1, hexokinase1, hsp75, and truncated-Bid (t-Bid), which also co-regulate the translocation of cytochrome c into the cytosol [37]. Therefore, the effect of Pirh2 on these proteins was also estimated in STZ and Aβ_1–42_ treated N2A cells. Silencing of Pirh2 in neuronal cells significantly inhibited the altered protein level of VDAC1, Hexokinase1, Hsp75, and t-Bid (Fig. 5a–j). In parallel, we also observed the immunofluorescence of VDAC1 in scrambled and Pirh2-silenced N2A cells. STZ or Aβ_1–42_ treatment caused significantly augmented levels of VDAC1 along with reduced mitotracker deep red uptake in STZ or Aβ_1–42_ treated N2A cells, which showed the affected mitochondrial functions. Nevertheless, transient silencing of Pirh2 in neuronal cells reduced the VDAC1 level and enhanced the mitotracker deep red uptake in neuronal cells (Fig. 5k–m).

### Mitochondrial protein cytochrome c interacts with Pirh2

**Fig. 6:**
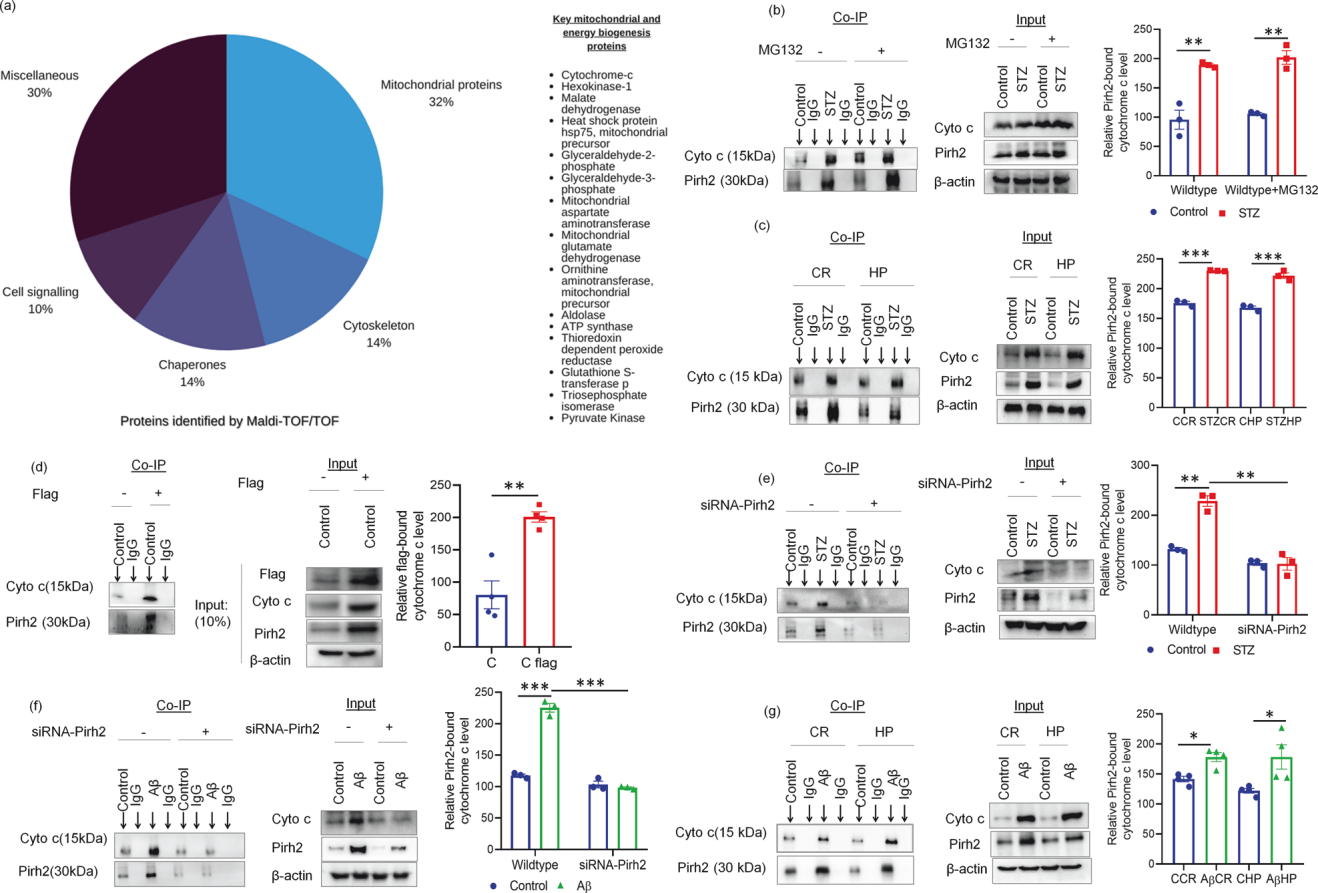
Pirh2 interacts with cytochrome c. **a** A pie chart of identified Pirh2 interacting proteins grouped by their functions, *n*_exp._ = 3. **b**, **c** Representative images of immunoblots illustrating the co-immunoprecipitation (Co-IP) of samples (N2A and rat brain CR and HP) with anti-Pirh2 monoclonal antibodies and IgG antibodies and probed for cytochrome c and Pirh2 in cell treated with or without STZ & MG132 and in rat brain with or without STZ administration *n*_exp._ = 3. **d** Representative images of Immunoblots illustrating the co-immunoprecipitation (Co-IP) in N2A cells of ectopically expressed Pirh2 using anti-flag antibodies and IgG antibodies and probed for cytochrome c and Pirh2 against IgG as a negative control in wildtype and (Myc-DDK-tagged)Pirh2 transient transfected N2A cells *n*_exp._ = 3. **e**, **f** The experiment was done in cytosolic fractions of different treatment sets therefore, cells were divided into two fraction one for confirming the Pirh2 silenced condition, and other part of cells cytosolic and mitochondrial fraction were isolated and Co-IP was performed. Images of immunoblots illustrating the co-immunoprecipitation (Co-IP) of endogenous cytochrome c and Pirh2 proteins using anti-Pirh2 antibodies and IgG antibodies in cytosolic fractions in scrambled and Pirh2 silenced N2A cells with or without STZ or Aβ treatment; *n*_exp._ = 3 and input blots of Pirh2 in whole cell lysate showing the conformation of Pirh2 silenced condition in STZ and Aβ treated N2A cells *n*_exp._ = 3. **g** Representative images of Immunoblots of co-immunoprecipitation (Co-IP) of cytosolic samples in rat brain CR and HP with anti-Pirh2 and IgG antibodies and probed for cytochrome c & Pirh2 proteins against IgG as a negative control with or without Aβ treatment; *n*_exp._ = 3, and input blot showing the expression of Pirh2 and cytochrome c in cytosolic fractions after 21 days post injection of Aβ in rat brain; *n*_exp._ = 4. All the data are represented as mean ± SEM, and *P* values were calculated with statistical test two-way ANOVA, followed by Tukey’s test. *p < 0.05, **p < 0.01, ***p < 0.001 control vs. STZ ; control MG132 vs. STZ MG132; scrambled control vs. scrambled (STZ or Aβ); scrambled (STZ or Aβ) vs. siRNA-Pirh2 (STZ or Aβ); Control cortex vs. STZ cortex; Control HP vs. STZ HP; Control cortex vs.Aβ cortex; Control HP vs. Aβ HP. C control, STZ streptozotocin, Aβ oligomeric Aβ_1–42_, Cyto c cytochrome c, DDK Flag, scrambled control siRNA, CR cortex, HP hippocampus.

Our above findings of mito-energetic impairment in AD conditions are in agreement with previous findings [56]. Further, we intend to investigate the mechanistic role of Pirh2 particularly by interactome study. To accomplish this, the MALDI-TOF (matrix-assisted laser desorption ionization-time of flight) based mass spectrometric analysis was done in both cell and tissue lysate, to find the interacting proteins of Pirh2 in control and treated conditions. For the same, the co-immunoprecipitation was performed using Pirh2 antibody in control and treated condition with or without MG132 (proteasome inhibitor) in N2A cells lysate and tissue lysates of cortex and hippocampus regions. The immunoprecipitated samples were then run for MALDI-TOF/TOF identified and characterized through MASCOT software (Figs. S4 & S5a, b). Interestingly, data showed the interaction of Pirh2 with various proteins that regulate mitochondrial functioning (Fig. 6a). However, based on our previous finding of cytochrome c translocation during AD pathology [39] and being it as a prime regulatory factor in mitochondria-mediated apoptotic signaling [57–59], the study was focused on cytochrome c. In order to further verify the MS data, the co-immunoprecipitation was performed using specific and relative antibody pulldown and immune-complexes were analyzed by immunoblotting along with IgG negative control. In concurrence, the high interaction of Pirh2 was found with cytochrome c, which was further considerably augmented in STZ treatment (Fig. 6b, c). In a reverse approach, the cell and tissue lysates were immunoprecipitated with cytochrome c and IgG antibodies, and samples were immunoblotted for Pirh2 and cytochrome c. Immunoblot images and analysis suggest that cytochrome c interact with Pirh2 in both N2A cells and in cortex and HP rat brain region (Fig. S6a, b). Altogether, both approaches confirm the high interaction of Pirh2 with mitochondrial protein cytochrome c in N2A cells and cortex and HP rat brain region. Moreover, the co-immunoprecipitation was also performed in myc-DDK-Pirh2 transient transfected N2A cells using Flag antibody and IgG antibody, and immune-complexes were analyzed by immunoblotting along with IgG negative control. This approach also confirmed that Pirh2 interacts with cytochrome c (Fig. 6d). To assess the interaction site for Pirh2 and cytochrome c the Co-IP was performed in both cytosolic and mitochondrial fractions. Data suggested that both Pirh2 and cytochrome c interact in cytosol. However, in siRNA-Pirh2 transfected N2A cells with or without STZ or Aβ_1–42_ treatment, a significant decrease in interaction was observed compared to scrambled control or STZ or Aβ_1–42_ treated N2A cells, further confirming that Pirh2 interacts with cytochrome c (Fig. 6e, f). Moreover, Co-IP was also performed in cellular fraction of control and Aβ_1–42_ administrated rat brain region in cortex and HP region of SD rat. We observed that the interaction was taking place in cytosolic fraction (Fig. 6g), while in mitochondrial fraction, no interaction was observed (data not shown). Taken together, all the approaches confirmed that Pirh2 interacts with cytochrome c.

### Cytochrome c is ubiquitylated by Pirh2 and proteasome activity in AD conditions

**Fig. 7:**
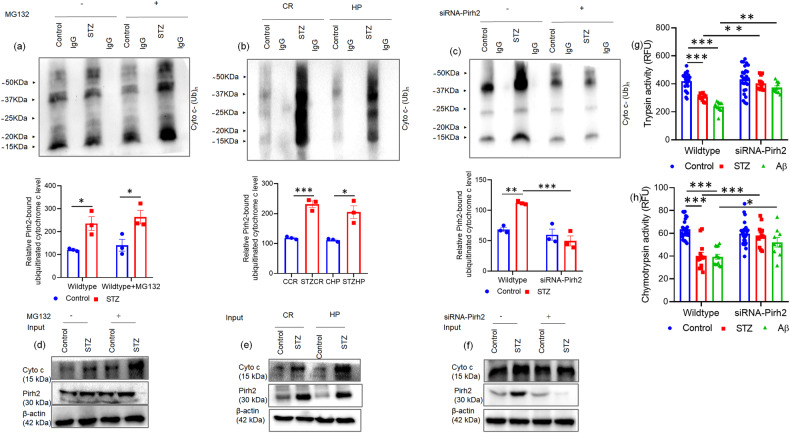
Pirh2 is involved in ubiquitylation of cytochrome c and proteasome activity. Representative image and graph of in vitro ubiquitylation assay with E2 enzyme UbcH13 after Co-IP using Pirh2 and IgG antibodies for endogenous ubiquitinated cytochrome c proteins in **a** N2A cell with or without MG132 and STZ treatment, **b** brain lysate of cortex and HP rat brain regions in control and 21 days post injection of STZ and **c** N2A cells after silencing with siRNA-Pirh2 and STZ treatment; *n*_exp_. = 3. Immunoblots represent the level of cytochrome c and Pirh2 in **d** N2A cells treated with MG132 and STZ and **e** in cortex and HP region of control and 21 days post injection of STZ in rat brain and **f** in N2A cells transfected with or without siRNA-Pirh2 and STZ treatment and β-actin used as loading control *n*_exp_. = 3. **g**, **h** Graphical representation for proteasome (trypsin, scrambled control *n* = 23; scrambled STZ *n* = 11; scrambled Aβ *n* = 10; siRNA-Pirh2 control *n* = 26; siRNA-Pirh2 STZ *n* = 12; siRNA-Pirh2 Aβ *n* = 10; from three independent experiment and in chymotrypsin, scrambled control *n* = 26; scrambled STZ *n* = 13; scrambled Aβ *n* = 13; siRNA-Pirh2 control *n* = 26; siRNA-Pirh2 STZ *n* = 14; siRNA-Pirh2 Aβ *n* = 9; from three independent experiments) activity in scrambled and Pirh2 silenced N2A cells with or without STZ or Aβ treatment. Data of **a**, **c** are represented as mean ± SEM, and statistical analysis were performed using two-way ANOVA, followed by Tukey’s test. *p < 0.05, **p < 0.01, ***p < 0.001 control vs. STZ ; control + mg132 vs STZ +mg132; scrambled STZ vs. siRNA-Pirh2 STZ. Data of **b** are represented as mean ± SEM and analyzed by unpaired two-tailed student’s *t* test, *p < 0.05, ***p < 0.001, Control vs. STZ. Data of **g**, **h** are represented as mean ± SEM and statistical analysis were performed using two-way ANOVA, followed by Tukey’s test. *p < 0.05, **p < 0.01, ***p < 0.001 scrambled control vs. scrambled (STZ or Aβ), scrambled (STZ or Aβ) vs. siRNA-Pirh2 (STZ or Aβ). STZ streptozotocin, Cyto c cytochrome c, scrambled control siRNA.

Being E3 ligase property of Pirh2 next, we checked the role of Pirh2 in the ubiquitylation of cytochrome c utilizing an in vitro ubiquitylation assay. Of various E2 enzymes used in the assay, we obtained considerable ubiquitylation of cytochrome c in the presence of E2 enzyme UbcH13 which is widely known to attach the K63-mediated ubiquitin chain on the target substrate. Our data suggest that Pirh2 ubiquitylated the cytochrome c in both control and STZ-treated conditions, and in transiently siRNA-Pirh2 transfected cells the ubiquitylation of cytochrome c was lesser, suggesting the involvement of Pirh2 in cytochrome c ubiquitylation (Fig. 7a–c). In coherence with immunoprecipitation results, the intensity of ubiquitylation was apparently higher in the STZ-treated group in comparison to the respective control set. The blot shows the protein abundance of Pirh2 and cytochrome c in respective sets of samples (Fig. 7d–f).

Further, the neuronal cells were transiently transfected with scrambled and siRNA-Pirh2, and proteasome activity was estimated in both wild-type and transfected cells with or without STZ or Aβ_1–42_ treatment. Both trypsin and chymotrypsin activity were measured utilizing trypsin substrate (Z-ARR-AMC) and chymotrypsin substrate (SUC-LLVY-AMC). Data showed that transient silencing of Pirh2 with siRNA-Pirh2 followed by STZ or Aβ_1–42_ treatment in N2A cells significantly attenuates the depleted proteasome activity (Fig. 7g, h).

Collectively findings suggested that Pirh2 participates in the ubiquitylation of cytochrome c and is also involved in proteasome function during AD pathogenesis.

### Role of Pirh2 in AD-related apoptotic signaling and DNA damage

Finally, the role of Pirh2 was elucidated in AD-related apoptotic signaling and DNA damage.

**Fig. 8:**
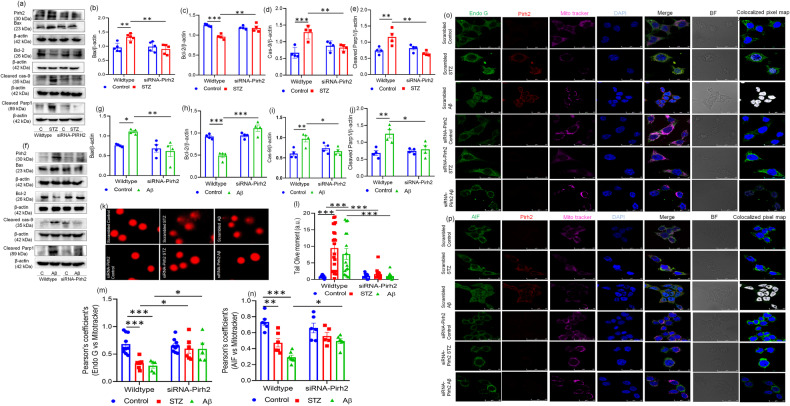
Effect of Pirh2 on mitochondrial-mediated neuronal cell death. **a**–**j** Immunoblots and graphical illustrations represent the protein level of Bax, Bcl-2, cleaved Caspase-9, and cleaved Parp1 in scrambled and siRNA-Pirh2 transfected N2A cells normalized against β-Actin to assess the effect of Pirh2 silencing upon STZ or Aβ treatment in comparison to the control, *n*_exp._ = 4–5. **k** Images showing the effect of Pirh2 silencing on DNA fragmentation by comet assay and **l** graphical representation of olive tail moment in scrambled and siRNA-Pirh2 transfected N2A cells with or without STZ treatment; (scrambled control *n* = 17; scrambled STZ *n* = 21; scrambled Aβ *n* = 16; siRNA-Pirh2 control *n* = 17; siRNA-Pirh2 STZ *n* = 17; siRNA-Pirh2 Aβ *n* = 17 are individual samples from three independent experiment (*n*_exp_ = 3) and analyzed by comet score 2 software. **m**, **n** Graph showing the Pearson’s coefficients between Endo-G with mitotracker deep red (scrambled control *n* = 10; scrambled STZ n = 8; scrambled Aβ *n* = 5; siRNA-Pirh2 control *n* = 9; siRNA-Pirh2 STZ *n* = 6; siRNA-Pirh2 Aβ *n* = 5, are individual samples from three independent experiment) and AIF with mitotracker deep red (scrambled control *n* = 6; scrambled STZ *n* = 6; scrambled Aβ *n* = 7; siRNA-Pirh2 control *n* = 6; siRNA-Pirh2 STZ *n* = 5; siRNA-Pirh2 Aβ *n* = 6, are individual samples from three independent experiment) in scrambled and siRNA-Pirh2 transfected N2A cells with or without STZ or Aβ treatment and analyzed by Fiji software. **o**, **p** Confocal microscopy images represent the translocation of endonuclease G & AIF from the mitochondria to nucleus along with Pirh2 (double-immunofluorescence) in scrambled and siRNA-Pirh2 after STZ or Aβ treatment in N2A cells in the presence of mitotracker deep red and DAPI counterstaining representing mitochondrion and nucleus, respectively; *n*_exp._ = 3 (Scale bar, 25 µm). Data are represented as mean ± SEM, and statistical analysis was performed using two-way ANOVA, followed by Tukey’s test. *p < 0.05, **p < 0.01, ***p < 0.001 scrambled control vs. scrambled (STZ or Aβ), scrambled (STZ or Aβ) vs. siRNA-Pirh2 (STZ or Aβ). CControl, STZ streptozotocin, Aβ Aβ(1–42) oligomer, scrambled control siRNA, AIF apoptosis-inducing factor, endo-G endonuclease G.

Previously, we have reported significant DNA damage in AD pathology in neuronal cells as well as in both cortex and hippocampus brain regions of the experimental rat model [35]. Therefore, the protein level of pro-apoptotic bax and anti-apoptotic protein bcl-2 was estimated in both scrambled and siRNA– Pirh2 with STZ or Aβ_1–42_ treated N2A cells. Interestingly, data showed that in siRNA-Pirh2 transfected neurons, the level of Bax was decreased significantly compared to only scrambled STZ or Aβ_1–42_ treatment in cells (Fig. 8a, b, f, g). Moreover, treatment with STZ or Aβ_1–42_ caused depleted level of bcl-2, which was inhibited in siRNA-Pirh2 transfected N2A cells (Fig. 8a, c, f, h). We have also measured the level of cleaved caspase-9, which is a downstream apoptotic factor of cytochrome c and an upstream factor of cleaved caspase-3. The STZ or Aβ_1–42_ induced increased protein level of cleaved caspase-9 was decreased after siRNA-Pirh2 transfection in neuronal N2A cells with STZ or Aβ_1–42_ treatment (Fig. 8a, d, f, i).

Additionally, DNA damage was also estimated as one of the markers of apoptosis through comet assay in both wild-type and Pirh2 silenced neuronal cells. Significantly increased DNA damage was observed in STZ or Aβ_1–42_ treated cells compared to control N2A cells. Data suggested significantly increased olive tail moment in STZ or Aβ_1–42_ treated N2A cells measured by comet score 2 software. However, in siRNA-Pirh2 transfected cells with or without STZ or Aβ_1–42_ treatment, significant inhibition in DNA damage was observed (Fig. 8k, l). DNA damage further caused the activation of PARP1 therefore, we measured the PARP1 level through immunoblotting and immunofluorescence in both scrambled and siRNA-Pirh2 transfected cells with or without STZ or Aβ_1–42_ treatment in N2A cells. Significantly increased level of cleaved PARP1 was observed in STZ or Aβ_1–42_ treated neuronal N2A cells, which was significantly decreased in siRNA-Pirh2 transfected cells with STZ or Aβ_1–42_ treatment (Fig. 8a, e, f, j). Additionally, immunofluorescence images also showed the increased labeling of PARP1 in STZ and Aβ_1–42_ treatment which was inhibited in siRNA-Pirh2 transfected cells with STZ or Aβ_1–42_ treatment in N2A cells (Fig. S7). The apoptotic inducing factor (AIF) and endonuclease G (endo-G) are the major proteins translocated from the mitochondria to the nucleus to induct the nuclear apoptotic signaling. Therefore, the translocation of both proteins was studied. Findings suggested that the endo-G and AIF were significantly translocated in STZ or Aβ1–42 treated N2A cells (Fig. 8m, n). Transient transfection of Pirh2 with siRNA-Pirh2 prevented the translocation of endo-G into the nucleus in STZ or Aβ_1–42_ treated neuronal N2A cells (Fig. 8m, o). Moreover, in siRNA-Pirh2 transfected cells treated with or without Aβ_1–42_ treatment, significant attenuation in the translocation of AIF into the nucleus was observed (Fig. 8n). However, in STZ-treated N2A cells, no significant prevention in the translocation of AIF was observed (Fig. 8n). In both, STZ or Aβ_1–42_ treatment the translocation of endo-G was observed which may be because endo-G participates in cleavage of both high molecular-weights DNA as well as oligonucleosomal DNA and forms complex with DNase-I & exonuclease to execute DNA processing while AIF induces the DNA fragmentation of DNA weighing 50 kbp along with chromatin condensation.

The difference in observation might also be due to varied mechanisms of action of utilized toxins to induce the diseased conditions. Observations suggested that silencing of Pirh2 more profoundly alters endo-G-mediated induction of nuclear fragmentation, compared to AIF-mediated DNA fragmentation.

Therefore, data suggested that Pirh2 participates in the activation of apoptotic proteins and induction of DNA damage in STZ and Aβ_1–42_ treated N2A cells primarily involving endo-G translocation.

## Discussion

Despite the cardinal role of protein aggregation-mediated neuronal death during AD pathology and the regulatory role of E3 ubiquitin ligases in protein degradation mechanisms like UPS, the findings are scarce in context to the role of various E3 ubiquitin ligases in AD pathogenesis. It has been suggested that UPS dysfunction drives AD pathology and is well associated with its pathological hallmarks like amyloid-β accumulation and Tau hyperphosphorylation [60, 61]. In view of the regulatory role of E3 ubiquitin ligases in UPS machinery, the dysfunction or changes in their level may propagate the disease-related aberrant processes and accelerate the AD pathology and, therefore, suggested to be a therapeutic target for the management of AD [62]. A recent report has compiled the E3 ubiquitin ligases explored in AD pathology [10] wherein the expression of NEDD4-1, MARCH8, RNF182 is upregulated in diseased conditions while the level of TTC3, Ube3A, CHIP, HRD1, and Parkin gets downregulated.

Further, some of the E3 ubiquitin ligases like Itch and TRAF6 get activated and may further initiate the neurodegenerative signaling mechanisms. In spite of recent reports and the known more than six hundred E3 ubiquitin ligases, the wide scope remains to explore their role in AD pathology. In view of the considerable contribution of DNA damage in AD pathology [50, 63] and the central role of Pirh2 in the regulation of DNA damage [64], the present study was conducted to investigate the role of Pirh2 in AD pathogenesis. Primarily, we targeted to estimate the alteration in the level of Pirh2 during the experimental model of AD and assessed its particular effect on AD-specific disease markers employing neuronal cells and experimental sporadic rodent model, as approximately ninety percent of cases are of sporadic type [65]. STZ or Aβ_1–42_ treatment in neuronal N2A cells and cortex & hippocampal regions of rat brain exhibited the significantly augmented level of Pirh2 along with Alzheimer’s disease markers like upregulated p-Tau and β-amyloid, increased AChE activity and neuronal apoptosis. siRNA-mediated silencing of Pirh2 with STZ or Aβ_1–42_ treatment inhibited the augmented level of the disease-specific pathological markers and implicated the particular role of Pirh2 in disease pathogenesis. The utilized N2A cells are cholinergic in nature, and treatment of STZ or Aβ_1–42_ exhibited the occurrence of AD-specific markers as reported previously by us and others [39, 66], therefore mimicking the diseased conditions in in vitro conditions. We have also assessed the protein level of Pirh2 and its effect on neuronal communication in differentiated neurons and observed the crucial role of Pirh2 in STZ or Aβ_1–42_ induced diseased conditions [67]. Further, in vivo findings are in concurrence and therefore suggested that the findings could be replicated in the brains of AD patients however, the investigation in post-mortem brain and clinical studies may provide better ratiocination.

Further, the investigations were done to assess the implications of Pirh2 in disease-related neurodegenerative signaling with a prime focus on mitochondria-mediated neuronal death. In agreement, recently also, the central role of mitochondria and energy metabolism has been suggested in AD conditions which are primarily due to the high demand of energy by post-mitotic neurons [68]. It has been reported that AD conditions exhibit impaired metabolism, mitochondria-associated oxidative stress, disrupted mitochondrial dynamics, and possibly biogenesis along with impaired transport, collectively suggesting the prime role of mitochondrion in Alzheimer’s disease pathogenesis. In fact, it has been reported that mitochondrial functions can be negatively affected by β-amyloid [6, 69], and a recent report has suggested utilizing the measurement of mitochondrial functions as a disease biomarker [70]. Therefore, the implications of Pirh2 in mitochondrion functions are worthwhile to elucidate. Findings suggested the significant mitochondrial dysfunction in STZ and Aβ_1–42_ employed experimental models as evidenced by depleted mitochondrial complex-I activity and ATP level and are in concordance with the previous finding of us and others [35, 55]. In siRNA-Pirh2 transfected cells, the altered mitochondrial functions were inhibited, suggesting the role of Pirh2 in energy biogenesis. The Δ*ψ*_m_ was significantly decreased in STZ and Aβ_1–42_ treated N2A cells which contribute to the formation of mitochondrial transition pore [71] as evidenced by the significant release of cytochrome c in the cytosol and is in accordance with our previous report [39]. Transient silencing of Pirh2 significantly inhibited the depleted Δ*ψ*_m_ and translocation of cytochrome c from mitochondria to cytosol in neuronal N2A cells. Along with the central role of the mitochondrion in energy metabolism, its significant role in free radical generation has also been suggested [72, 73]. Therefore, further, the ROS generation, as well as the levels of GSH and MDA, were estimated. STZ or Aβ_1–42_ treatment in N2A cells caused oxidative stress as observed with augmented ROS generation and altered levels of GSH and MDA, which were significantly attenuated in Pirh2 silenced neuronal cells suggesting the contribution of Pirh2 in cellular antioxidant system during STZ or Aβ_1–42_ treatment condition in N2A cells.

The depleted Δ*ψ*_m_ induced formation of mitochondrial transition pore further enhances the expression of proteins located on the outer mitochondrial membrane, particularly VDAC1, which is considered a gatekeeper of mitochondria and regulates the mitochondrial apoptosis through bcl-2 family of proteins like t-Bid, bax, and hexokinase [74–80]. In fact, one of the recent studies showed the lineal interaction of VDAC1 and Bid in the maintenance of mitochondrial functions [81]. In this regard, we have observed the altered level of VDAC1, hsp75, t-Bid, and hexokinase1 in STZ or Aβ_1–42_ treated neuronal cells, which were inhibited in Pirh2 silenced neuronal cells. It has also been reported that hexokinase1 inhibits the VDAC1 conductance and exerts anti-apoptotic effects by preventing the release of cytochrome c to promote neuronal survival in physiological conditions [82]. Our finding of the depleted level of hexokinase1 in STZ or Aβ_1–42_ treated N2A cells is in agreement with the previous report and indicates its contribution to disease pathogenesis. Additionally, the observed decreased level of hsp75 suggests the cellular incapability to regulate the metabolic rate of ETC, switching from OXPHOS to glycolysis, thereby decreasing ROS [83]. Moreover, hsp75 is also involved in the maintenance of mitochondrial integrity through mitochondrial permeability transition pore opening, and thus depleted hsp75 contributes to pro-apoptotic signaling in STZ and Aβ_1–42_ treated N2A cells [84]. Depleted Δ*ψ*_m_ and alteration in other signaling proteins lead to the formation of mitochondrial transition pore, which allows the release of cytochrome c as observed in both employed experimental models. During physiological conditions, cytochrome c remains bound to cardiolipin, while in diseased conditions, it is solubilized and initiates apoptotic signaling through caspase-9 [85]. Concurrently, we have observed the augmented level of cleaved caspase-9 in STZ or Aβ_1–42_ treated neuronal N2A cells, which were impeded in Pirh2 silenced cells. In agreement with the observed effect of Pirh2 in mitochondrial functionality, further, we have investigated whether Pirh2 has an interacting capacity with mitochondrial proteins or not. MALDI-TOF/MS data suggested that a number of signaling proteins interacted with Pirh2 however, this study was focused on mitochondrion therefore, cytochrome c was selected further to investigate its mechanistic role in STZ or Aβ_1–42_ induced experimental model of AD. The MALDI-TOF/MS data was verified with co-immunoprecipitation, and a significant interaction of Pirh2 with cytochrome c was observed in both neuronal cells and studied rat brain regions. Since Pirh2 is a RING domain-containing E3 ubiquitin ligase, further we intended to elucidate its ubiquitylating capacity towards cytochrome c. Findings suggested that Pirh2 ubiquitylates the cytochrome c utilizing E2 enzyme UbcH13 which acts through lysine-63 residue. It has been reported that lysine-63 linked ubiquitylation directs the substrate proteins for proteasomal degradation [86], lysosomal degradation [87], and autophagy [88] however, its role in the ubiquitylation of protein kinases and phosphatases has also been reported [89]. Consistent with our aforementioned findings, impairment in ATP generation, in turn vitiates both UPS and proteasome activity, and therefore, the proteasome activity was also measured. STZ or Aβ_1–42_ treatment in neuronal N2A cells caused a reduction in proteasome activity, which was inhibited in Pirh2 silenced neurons suggesting the contribution of Pirh2 in proteasome-mediated protein degradation and needs to be studied further for details, particularly in the context of the role of deubiquitinating enzymes.

The diseased conditions induced translocated cytochrome c to form the complex with apaf-1 and caspase-9 to form the apoptosome and cause the cleavage of caspase-3 to induce neuronal apoptosis [37]. Therefore, the level of both cleaved caspase-9 and cleaved caspase-3 was estimated in STZ or Aβ_1_42 treated neuronal N2A cells. Pirh2 silenced cells showed less caspase-9 and cleaved caspase-3 level in STZ or Aβ_1–42_ treated N2A cells advocating the role of Pirh2 in neuronal apoptosis. Since mitochondrial transition pore formation may alter the level of both anti and pro-apoptotic proteins [90]. Therefore, the level of apoptotic factors was measured in N2A cells treated with STZ or Aβ_1–42_. STZ or Aβ_1–42_ treated neuronal cells showed a depleted level of bcl-2 and an augmented level of bax, which were inhibited in Pirh2 silenced neuronal cells. In fact, the mitochondrial dysfunction-induced release of cytochrome c further causes the caspase activation-induced apoptosis and DNA damage, which in turn enhances the release of cytochrome c from mitochondrion and worsens the conditions [91]. Therefore, DNA damage was also estimated in STZ or Aβ_1–42_ treated neuronal N2A cells. Findings showed significant DNA damage in STZ or Aβ_1–42_ treated neuronal N2A cells, which was significantly decreased in Pirh2 silenced cells suggesting the involvement of Pirh2 in DNA damage in employed experimental models. Additionally, DNA damage further caused the PARP1 activation. Therefore, we also checked the PARP1 level in STZ or Aβ_1–42_ treated neuronal N2A cells and Pirh2 silenced cells. Interestingly, we observed that in Pirh2 silenced cells with STZ or Aβ_1–42_ treatment, cleaved PARP1 level was significantly reduced, and observation is advocating that Pirh2 is involved in DNA damage in STZ or Aβ_1–42_ treated N2A cells.

Along with caspase-dependent cell death, depleted Δ*ψ*_m_ may also contribute to caspase-independent cell death, particularly through endonuclease G and AIF [92]. Both AIF and endonuclease G are translocated to the nucleus and contribute to DNA damage, as reported in AD pathology [63, 93]. Data showed that Pirh2 silenced neurons inhibited the translocation of endonuclease G from the mitochondria in STZ or Aβ_1–42_ treated neuronal N2A cells. However, AIF translocation was significantly inhibited in Aβ_1–42_ treated neuronal cells only, which might be due to varied mechanisms of action of utilized toxin and differed actions of endo-G and AIF, as endo-G cleaves both high molecular weight DNA as well as oligonucleosomal DNA and forms complex with DNase-I & exonuclease to execute DNA processing while AIF induces the DNA fragmentation of DNA weighing 50 kbp along with chromatin condensation [94, 95]. Findings also suggested the significant participation of Pirh2 in a caspase-independent pathway-mediated neuronal death.

## Conclusion

**Fig. 9:**
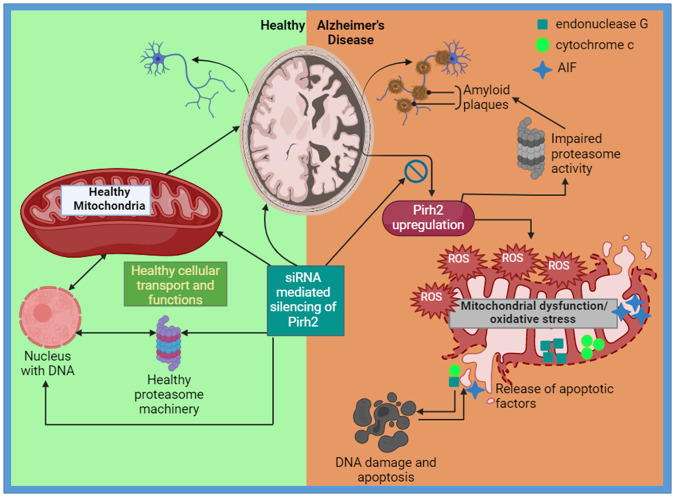
Diverse implications of Pirh2 in Alzheimer’s disease pathology. Findings of the study suggested the multiple target effect of pirh2 in AD pathology based on both cellular and rat models. However, there are several other known factors which are known to contribute in these neurodegenerative mechanisms therefore, the conflated investigation is still require to consider the Pirh2 as therapeutic target for the AD management.

In conclusion (Fig. 9), we demonstrated the crucial role of Pirh2 in mitochondrial functionality primarily through regulating the cytochrome c translocation, modulating the VDAC1 functioning and other mitochondrial proteins that participate in the maintenance of mitochondrial membrane potential and mitochondrial transition pore formation during STZ or Aβ_1–42_ induced AD pathogenesis. However, our study was specific to sporadic AD, and the limitation of this study is the non-availability of the Pirh2 knock-out rodent model to depict its effect on AD-linked critical proteins in in vivo models.

Nevertheless, this study highlights a particular effect of Pirh2 on AD-specific pathological markers in N2A cholinergic cell lines, which expressed high levels of Tau protein; therefore, for intracellular signaling of disease mechanism N2A is widely utilized in Alzheimer’s disease pathogenesis. Since cholinergic dysfunction and Tau protein are involved in AD pathogenesis, therefore, for this study, N2A is utilized. The findings reveal that Pirh2 silenced neuronal cells exhibited improved mitochondrial function, restored Δ*ψ*_m_, inhibition of cytochrome c translocation, restored proteasome activity, and prevented neuronal apoptosis. In the future, the investigation with advanced detection or imaging technologies, along with its alteration in the periphery during AD onset and progression, is required to develop Pirh2 as a diagnostic or therapeutic target in clinics.

### Supplementary information

Supplementary information

Original Data File

## Data Availability

All the generated data are compiled and given in MS. The raw data will be provided on reasonable request.
