# Supplementary material for: Pirh2 modulates the mitochondrial function and cytochrome c-mediated neuronal death during Alzheimer’s disease
Source: Cell Death Dis. 2024 May 13;15(5):331. doi: 10.1038/s41419-024-06662-1 (PMC11091053; doi:10.1038/s41419-024-06662-1)
Supplement: Supplementary file 1 — Supplementary information [file 41419_2024_6662_MOESM1_ESM.docx]

**Chemicals:**Bovine serum albumin (BSA), disodium hydrogen phosphate (Na_2_HPO_4_), dimethyl sulfoxide (DMSO), ethidium bromide, glucose,4-(2-hydroxyethyl)-1-piperazine ethane sulfonic acid (HEPES), 3-(4,5-dimethylthiazol-2-yl)-2,5- diphenyltetrazolium bromide dye (MTT), NP-40, phenylmethylsulfonyl fluoride (PMSF), magnesium chloride (MgCl_2_), dithiothreitol (DTT), RNase, sodium bicarbonate and tris-buffer were procured from SRL, India. Dulbecco's modified Eagle's medium (DMEM), fetal bovine serum (FBS), Trizol, Ham's F12 medium, penicillin–streptomycin, nuclease-free water, lipofectamine 3000 and mitotracker-deep red were purchased from Invitrogen (San Diego, CA, USA). *In-vitro* ubiquitylation kit was purchased from Enzo-Life sciences. Other chemicals such as Protein-G Sepharose beads, anti-fade medium DAPI, copper sulphate (CuSO_4_), calcium chloride (CaCl_2_), Folin–Ciocalteu reagent, potassium chloride (KCl), sodium carbonate (NaHCO_3_), sodium chloride (NaCl), sodium dihydrogen phosphate (NaH_2_PO_4_), sodium hydroxide (NaOH), protease and phosphatase inhibitor cocktail, streptozotocin, β-amyloid_1-42_ peptide. paraformaldehyde (PFA), ethylenediaminetetraacetic acid (EDTA), acetonitrile (ACN), ammonium bicarbonate (ABC), trifluoro acetic acid (TFA) and trypsin MS grade were obtained from Sigma, USA.

Table 1: Antibodies used for Immunoblot (WB) and immunofluorescence (IF)

| Antibodies | Company | Catalogue  Number | Species | Dilution  WB | Dilution  IF | Observed molecular weight kDa |
| --- | --- | --- | --- | --- | --- | --- |
| β-Actin | Sigma-Aldrich | A3854 | Mouse | 1:10000 | - | 42 |
| p-Tau | Abcam | ab151559 | Rabbit | 1:1000 | 1:100 | 50 |
| Tau | Invitrogen | 13-6400 | Mouse | 11000 | - | 50 |
| β-amyloid | Santa Cruz  Biotechnology | sc-28365 | Mouse | 1:500 | 1:100 | 4-50 |
| Caspase-3 | Invitrogen | 700182 | Rabbit | 1:1000 | - | 17 |
| Pirh2 | Santa Cruz  Biotechnology | sc-374505 | Mouse | 1:500 | 1:100 | 30 |
| VDAC1 | Cell Signalling | 4661S | Rabbit | 1:1000 | 1:100 | 32 |
| Hexokinase 1 | Cell Signalling | 2024 | Rabbit | 1:1000 | - | 102 |
| Bax | Santa Cruz  Biotechnology | sc-6236 | Rabbit | 1:500 | - | 23 |
| Bid | Santa Cruz  Biotechnology | sc-56025 | Mouse | 1:500 | - | 22 |
| Bcl-2 | Santa Cruz  Biotechnology | sc-7382 | Mouse | 1:500 | - | 26 |
| Cleaved- PARP-1 | Santa Cruz  Biotechnology | sc-56196 | Mouse | 1:500 | - | 89 |
| PARP-1 | Santa Cruz  Biotechnology | sc-8007 | Mouse | - | 1:100 | - |
| Endo-G | Santa Cruz  Biotechnology | sc-26924 | Goat | - | 1:100 | - |
| AIF1 | Santa Cruz  Biotechnology | sc-9416 | Goat | - | 1:100 | - |
| Caspase-9 | Santa Cruz  Biotechnology | sc-56076 | Mouse | 1:500 | - | 35 |
| HSP75 | Santa Cruz  Biotechnology | sc-390061 | Mouse | 1:1000 | - | 75 |
| Cytochrome c | Santa Cruz  Biotechnology | sc-13156 | Mouse | 1:500 | - | 15 |
| Cytochrome c | Cell Signalling | 11940 | Rabbit | - | 1:200 | - |
| Alexa-fluor 488 Green | Invitrogen | A11034 | Rabbit | - | 1:300 | - |
| Alexa-fluor  488 Green | Invitrogen | A11059 | Mouse | - | 1:300 | - |
| Alexa-fluor 488 Green | Invitrogen | A21467 | Goat | - | 1:300 | - |
| Alexa-fluor 532 Red | Invitrogen | A11002 | Mouse | - | 1:300 | - |
| Anti-Mouse Secondary | Sigma-Aldrich | A9044 |  | 1:5000 | - | - |
| Anti-Rabbit Secondary | Sigma-Aldrich | A0545 |  | 1:3000 | - | - |

**Supplementary Figure 1**


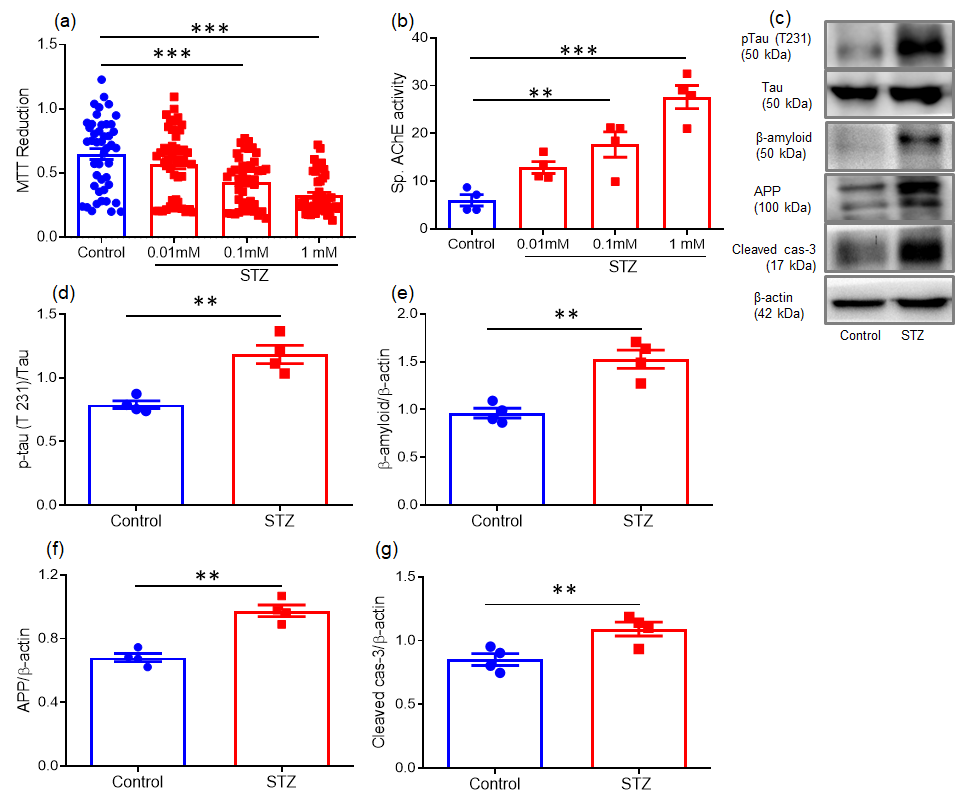


**Supplementary Figure 1- STZ induced AD specific pathological markers in N2A cells: (a)** Graphical representation for cell viability as estimated by mitochondrial dehydrogenase activity (MTT) in N2A cells treated with STZ for 48 hours, Control n=45; STZ (0.01mM, 0.1mM and 1mM) n=45 are individual samples from three independent experiments. Data was analyzed by one-way ANOVA followed by Dunnett’s post hoc test, ***p<0.001 Control vs. STZ. **(b)** Bar diagram showing the effect of STZ in AChE activity in N2A cells treated with STZ for 48 hours, n_exp._=4. Data are represented as mean± SEM and analyzed by one-way ANOVA followed by Dunnett’s post hoc test, **p<0.01, ***p<0.001 Control vs. STZ. **(c-g)** Immunoblots & graph indicating the alternation in AD specific pathological protein p-Tau, Tau, β-amyloid and amyloid precursor protein and neuronal apoptosis cleaved caspase-3 in N2A cells after treatment with STZ for 48 hours, n_exp._=4. Data are represented as mean± SEM and analyzed by unpaired two-tailed student’s t-test, **p<0.01, Control vs. STZ. Abbreviations: STZ-Streptozotocin, p-tau(T231)- phosphorylated tau at threonine 231, APP-Amyloid Precursor protein, n_exp._- number of independent experiment, n-number of individuals sample.

**Supplementary Figure 2**


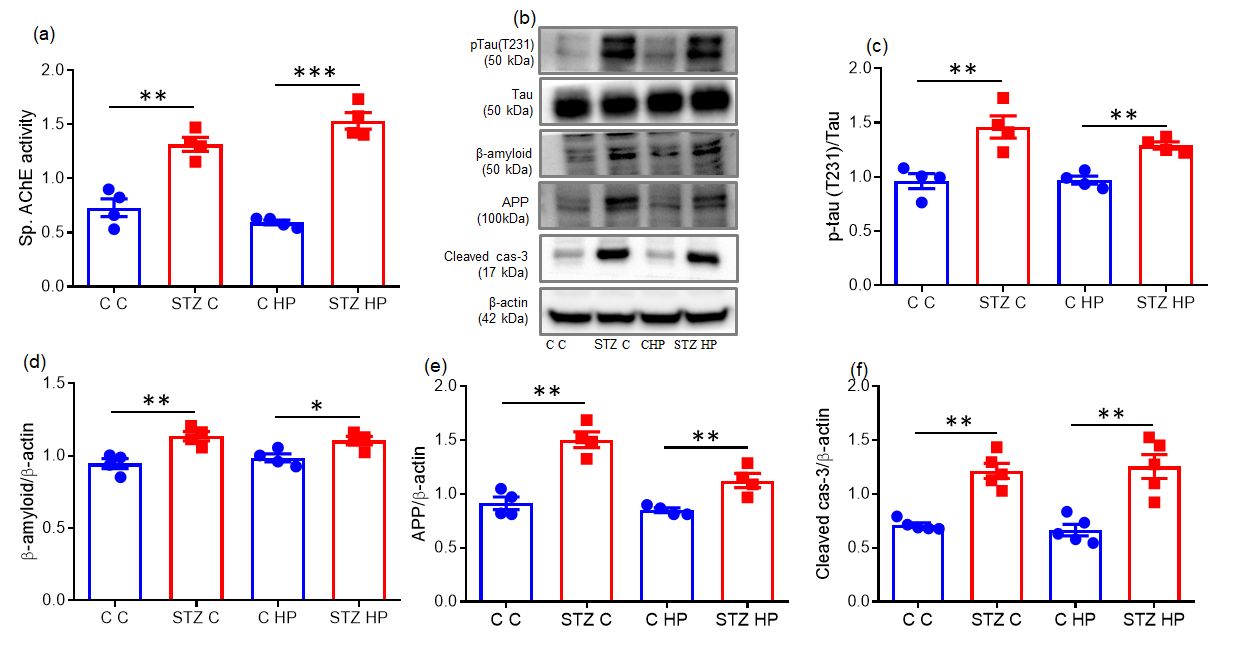


**Supplementary Figure 2- STZ induced AD specific pathological markers in rat brain: (a)** Bar diagram showing the effect of STZ in AChE activity in cortex and HP brain region, n_exp._=4. Data are presented as mean±SEM and statistically analyzed by unpaired two- tailed student’s t-test, **p<0.01, ***p<0.001 Control vs STZ. **(b-f)** Immunoblots & graphs showing the protein level of p-Tau, tau, β-amyloid, APP & cleaved caspase 3 in cortex and HP region of rat brain, n_exp._=4-5. Data are presented as mean±SEM and statistically analyzed by unpaired two-tailed student’s t-test, *p<0.05,**p<0.01, Control vs STZ;. Abbreviations: C C-control cortex; STZ C- streptozotocin cortex; STZ HP- streptozotocin hippocampus; C HP- control hippocampus; STZ-streptozotocin; C-control; p-tau(T231)- phosphorylated tau at threonine 231; APP-Amyloid Precursor protein.

**Supplementary Figure 3**


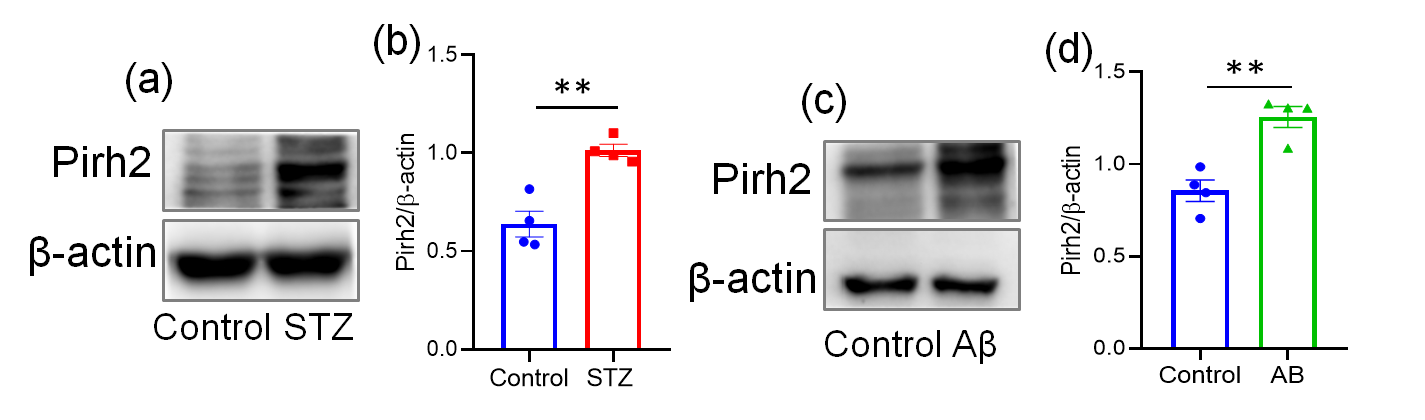


**Supplementary Figure 3- STZ and** **Aβ_1-42_ induced protein abundance of Pirh2 in SH-SY5Y cells: (a-d)** Immunoblots & graph showing the protein abundance of Pirh2 in SH-SY5Y cells after treatment with STZ for 48 hours and Aβ_1-42_ for 24 hours, n_exp._=4. Data are represented as mean± SEM and analyzed by unpaired two-tailed student’s t-test, **p<0.01, Control vs. STZ or Aβ_1-42_. Abbreviations: STZ-Streptozotocin, Aβ_1-42-_ oligomer Aβ_1-42._

**Supplementary Figure 4**


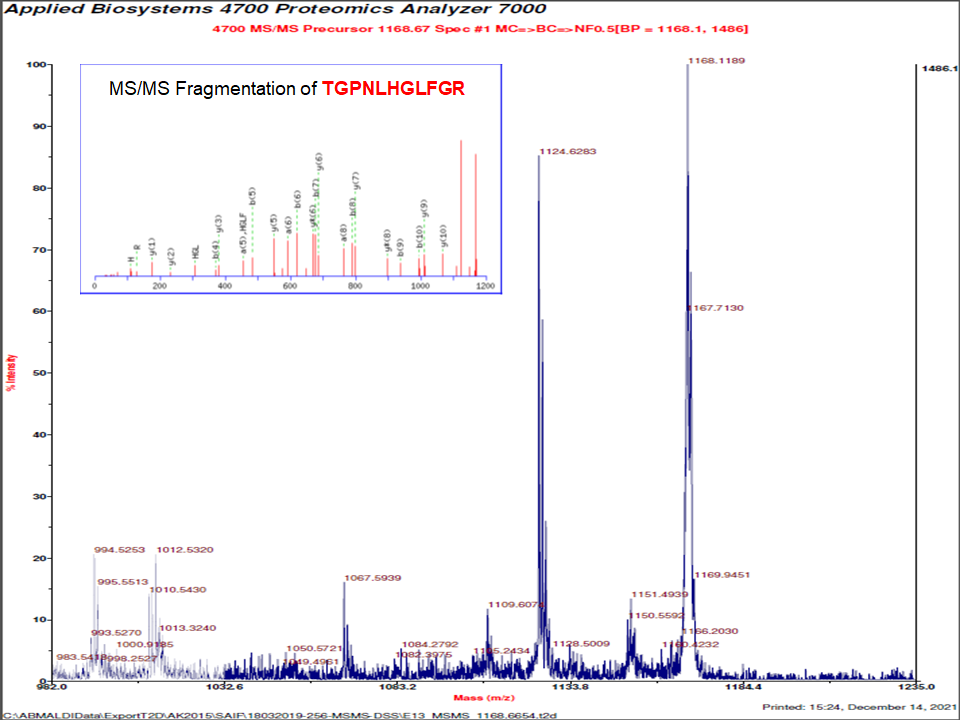


**Supplementary Figure 4-** MS/MS peaks representing the MS fragmentation spectrum (highest intensity) obtained from the tryptic peptides of amino acids (TGPNLHGLFGR) of cytochrome c in N2A cells, n_exp._=3.

**Supplementary Figure 5a**


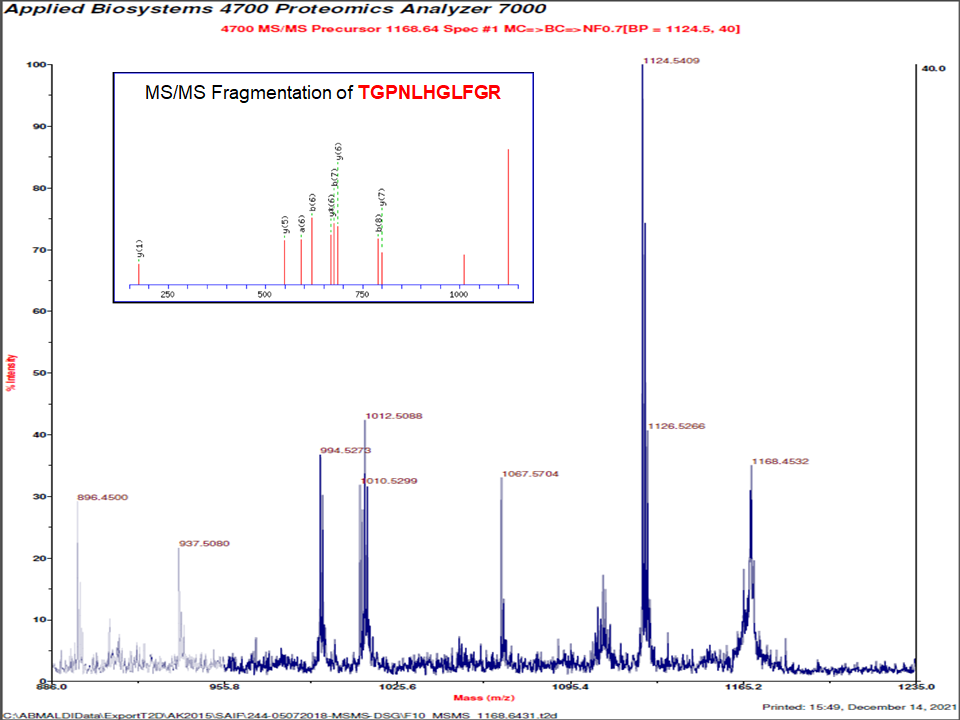


**Supplementary Figure 5b**


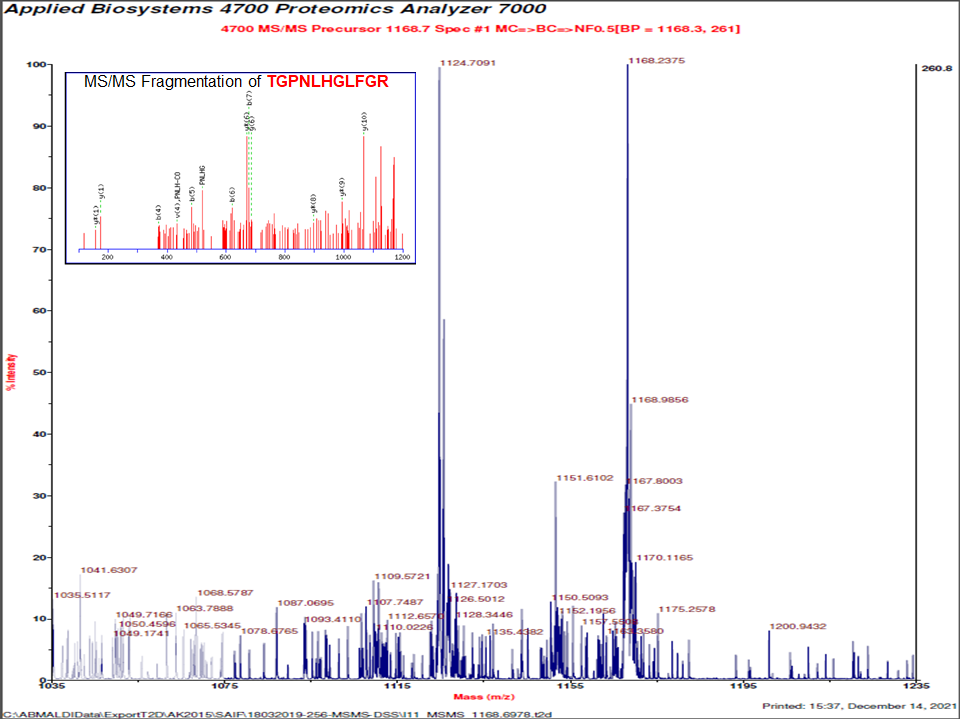


**Supplementary Figure 5- (a,b)** MS/MS peaks representing the MS fragmentation spectrum (highest intensity) obtained from the tryptic peptides of amino acids (TGPNLHGLFGR) of cytochrome c in rat brain regions of cortex and hippocampus respectively, n_exp._=3.

**Supplementary Figure 6**


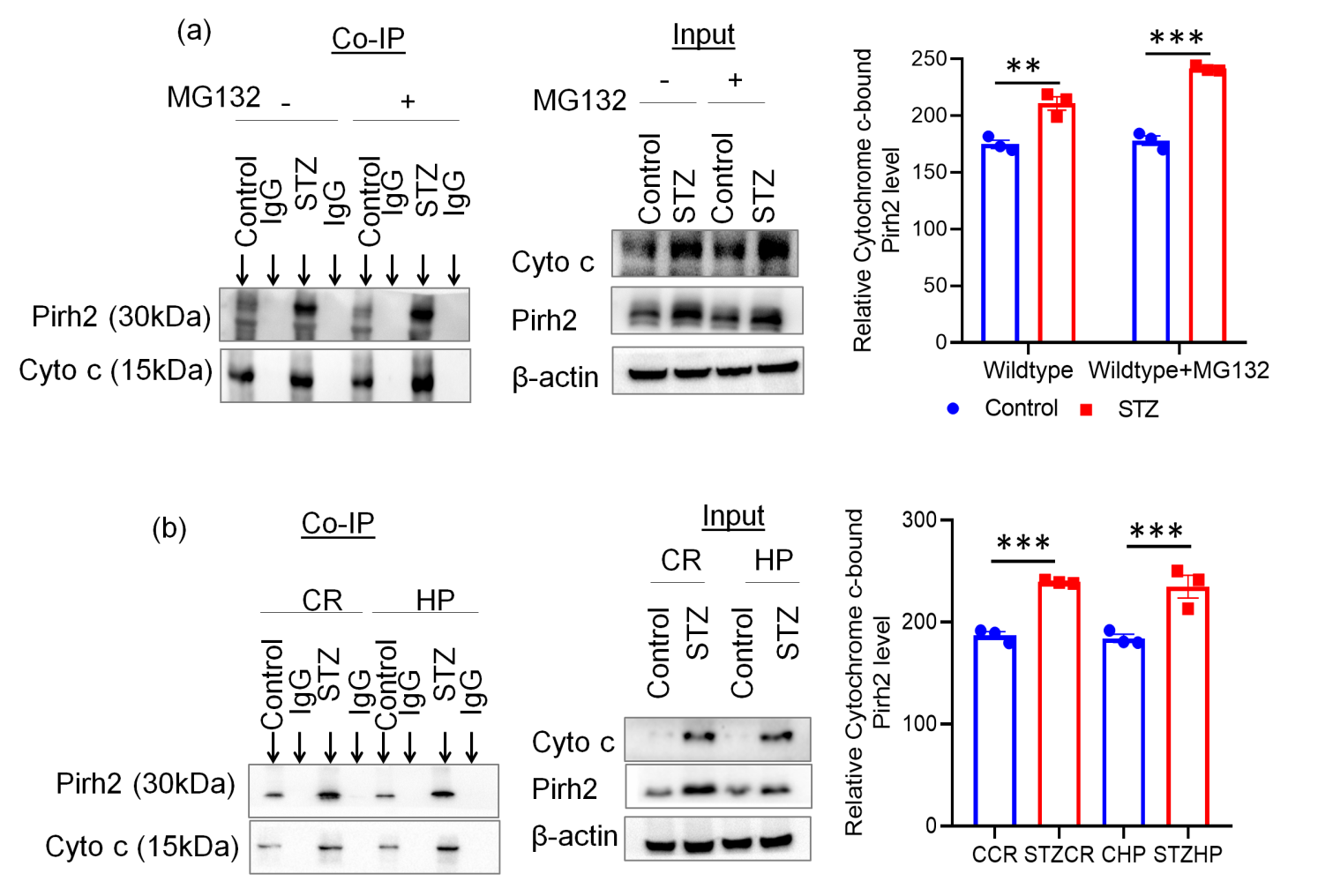


**Supplementary Figure 6- Cytochrome c interact with Pirh2: (a&b)** Representative images of blot illustrating the co-immunoprecipitation (Co-IP) of samples (N2A and rat brain CR and HP) with anti-cytochrome c monoclonal antibodies and IgG antibodies and probed for Pirh2 and cytochrome c against IgG as a negative control with or without STZ & MG132 treatment; n_exp._=3. Data are represented as mean±SEM and statistical analysis were performed using two-way ANOVA, followed by Tukey’s test.** p<0.01, *** p<0.001 control vs. STZ ; control MG132 vs. STZ MG132; Control cortex vs. STZ cortex; Control HP vs. STZ HP. Abbreviation: C- control, STZ-streptozotocin, Cyto c- cytochrome c, CR-cortex, HP-hippocampus.

**Supplementary Figure 7**


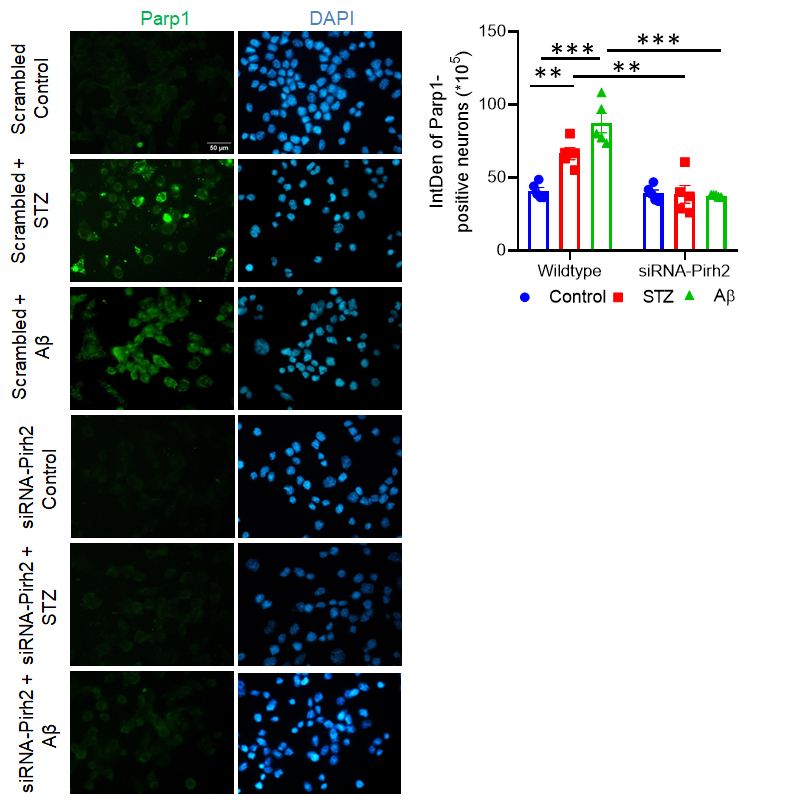


**Supplementary Figure 7- Effect of silencing of Pirh2 on PARP1 level in N2A cells:** Immunofluorescence images (40x) dipicating the expression of Parp1 (green) and counterstaining with DAPI (blue) in scrambled and siRNA-Pirh2 transfected N2A cells with or without STZ or Aβ treatment, n_exp._=3 (Scale bar, 50 µm). Graph showing the quantifications of Integrated density of Parp1 (scrambled control n=5; scrambled STZ n=5; scrambled Aβ n=5; siRNA-Pirh2 control n=5; siRNA-Pirh2 STZ n=5; siRNA-Pirh2 Aβ n=5 are individuals sample from three independent experiments) in neuronal N2A cells after transient transfection with scrambled and siRNA-Pirh2 with or without STZ and Aβ treatment. Quantifications are represented as mean±SEM and statistical analysis were performed using two-way ANOVA followed by Tukey’s test. ** p<0.01, *** p<0.001 scrambled control vs. Scrambled (STZ or Aβ); scrambled (STZ or Aβ) vs. siRNA-Pirh2 (STZ or Aβ). Abbreviation: STZ-streptozotocin; Aβ- Aβ(1-42) oligomer, scrambled- control siRNA.
