# Supplementary material for: Pirh2 modulates the mitochondrial function and cytochrome c-mediated neuronal death during Alzheimer’s disease
Source: Cell Death Dis. 2024 May 13;15(5):331. doi: 10.1038/s41419-024-06662-1 (PMC11091053; doi:10.1038/s41419-024-06662-1)
Supplement: Supplementary file 2 — Original Data File [file 41419_2024_6662_MOESM2_ESM.pptx]

## Slide 1
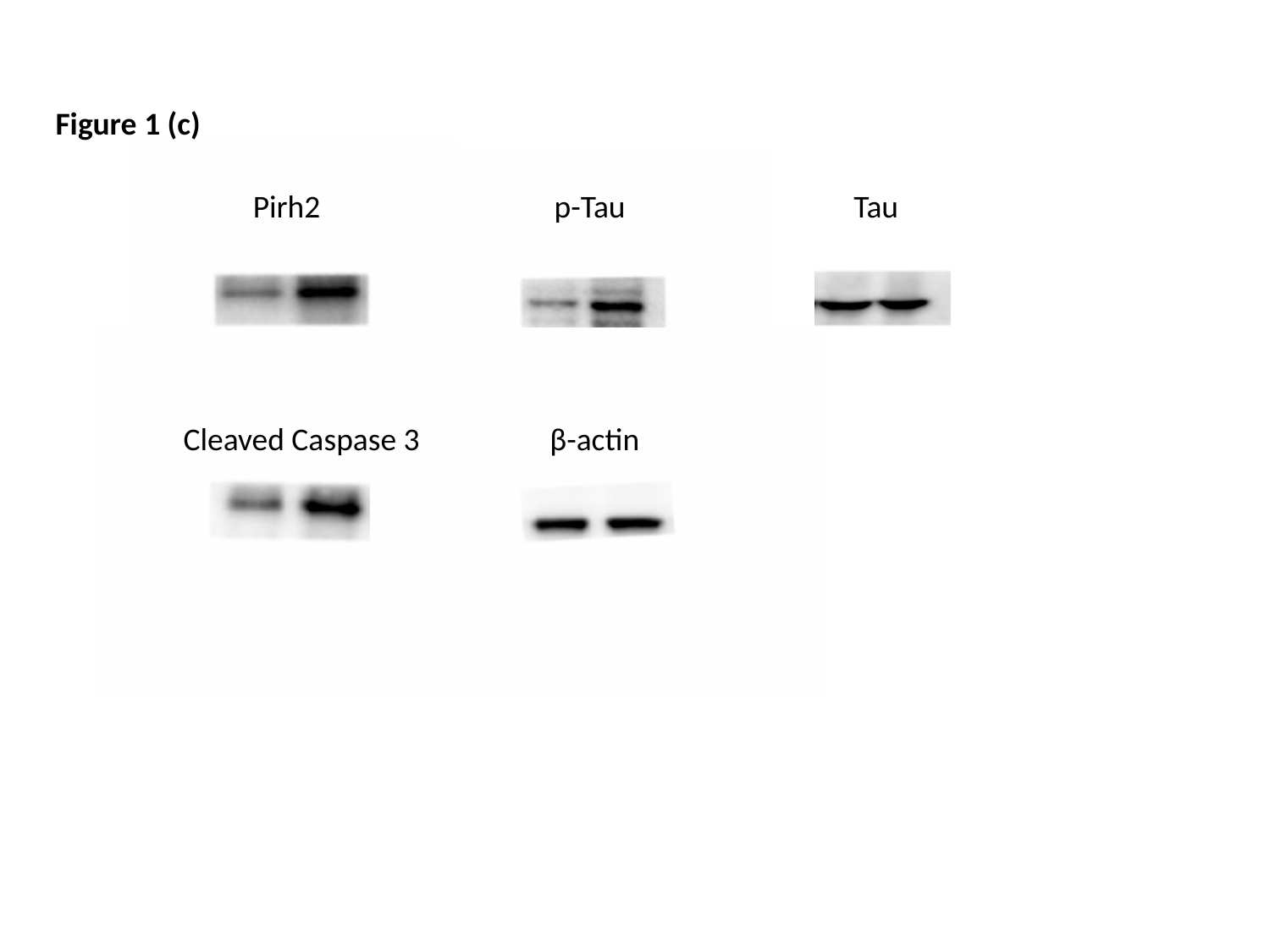

Figure 1 (c)
 Pirh2
 p-Tau
 Tau
Cleaved Caspase 3
 β-actin

## Slide 2
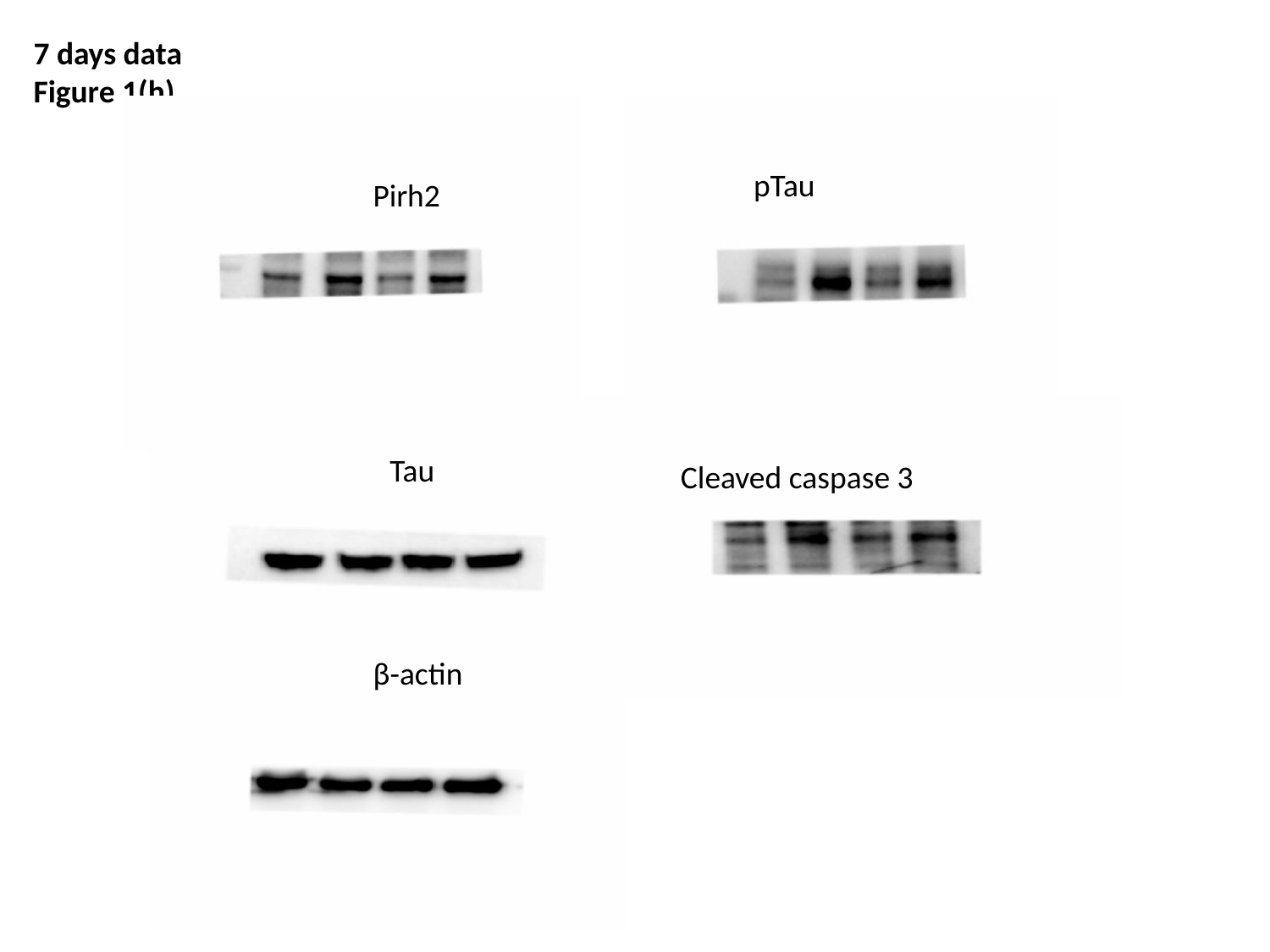

7 days data
Figure 1(h)
 pTau
Pirh2
Tau
Cleaved caspase 3
β-actin

## Slide 3
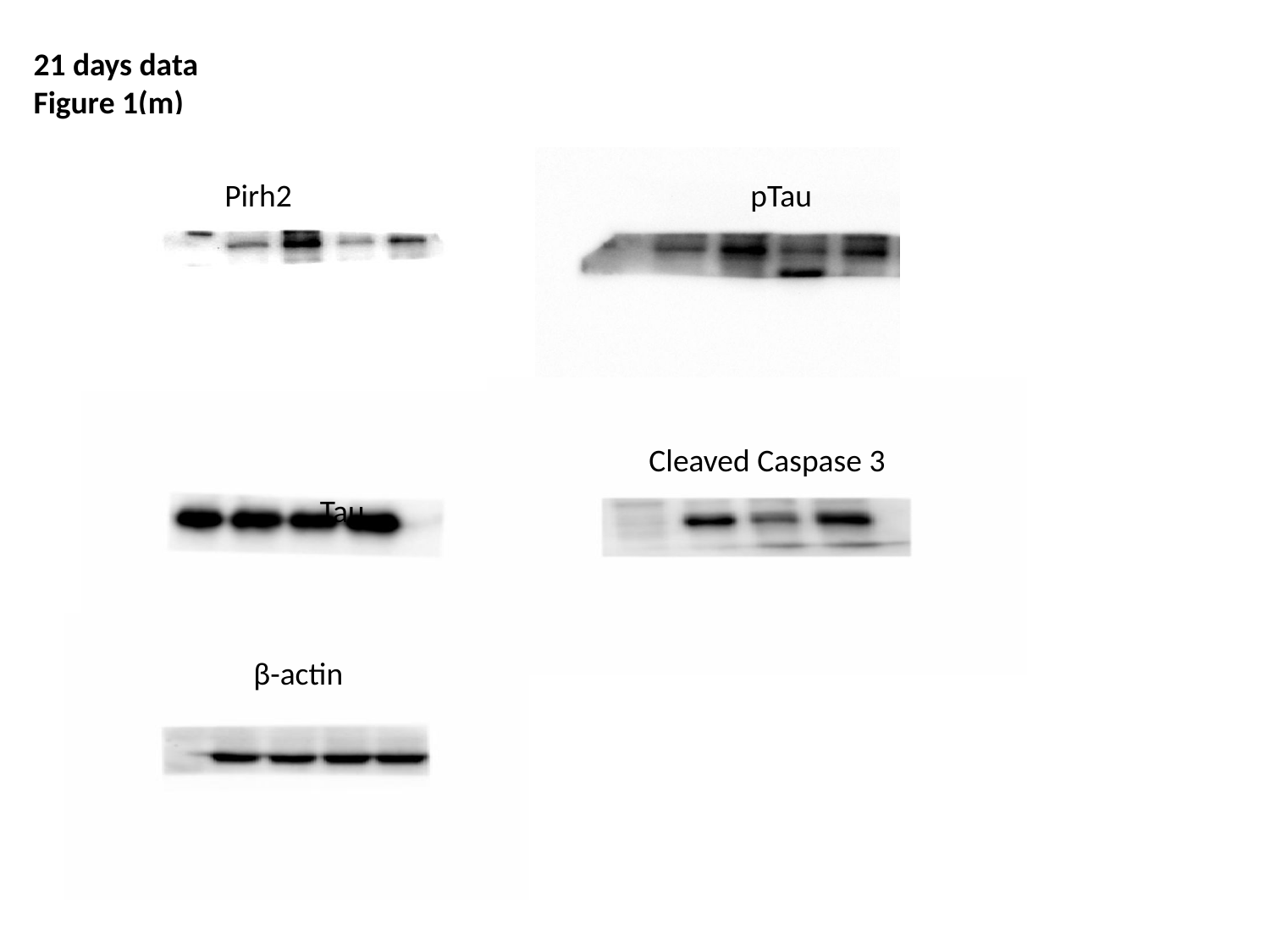

21 days data
Figure 1(m)
Pirh2
pTau
 Tau
Cleaved Caspase 3
 β-actin

## Slide 4
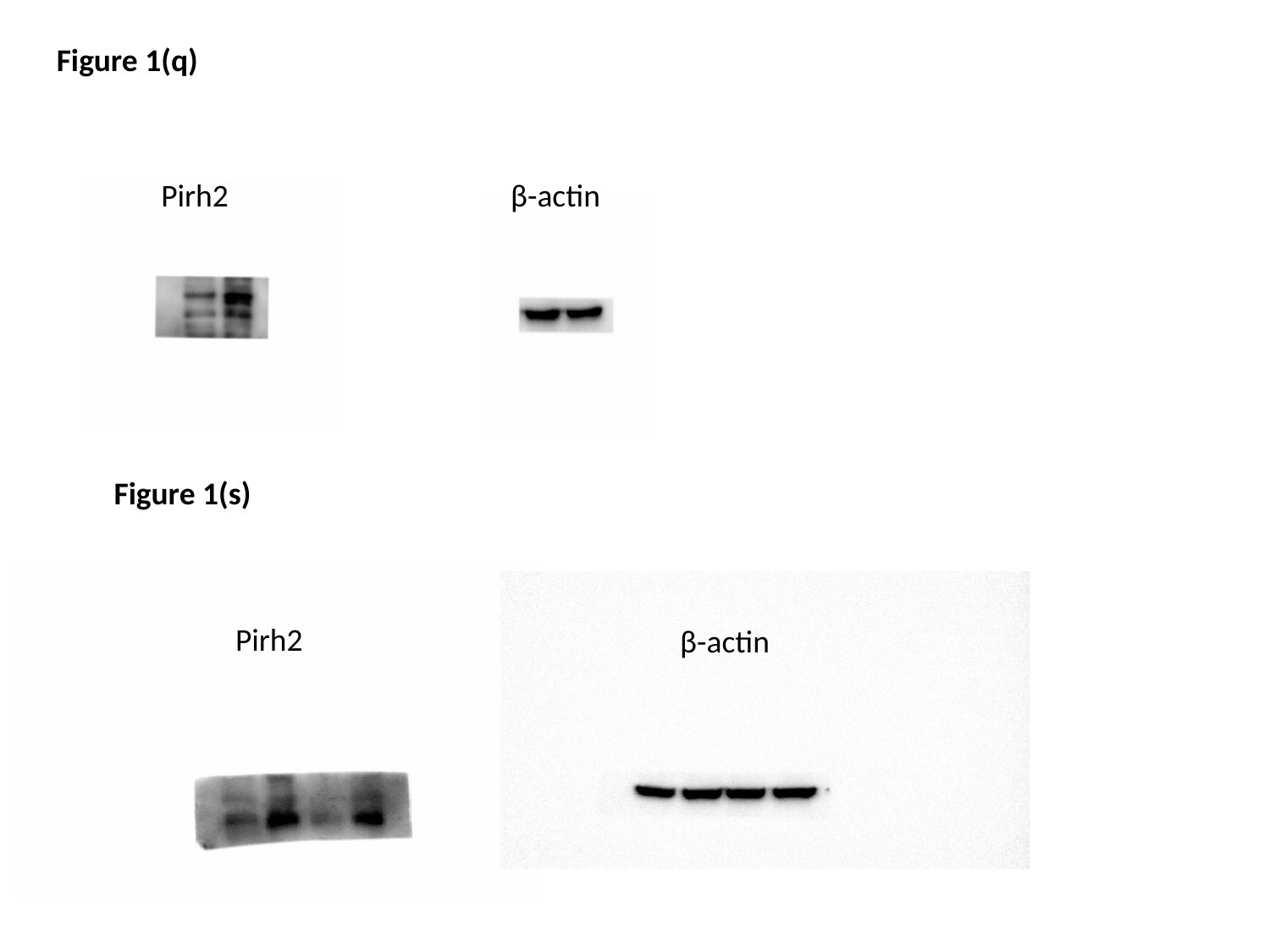

Figure 1(q)
Pirh2
β-actin
Figure 1(s)
Pirh2
β-actin

## Slide 5
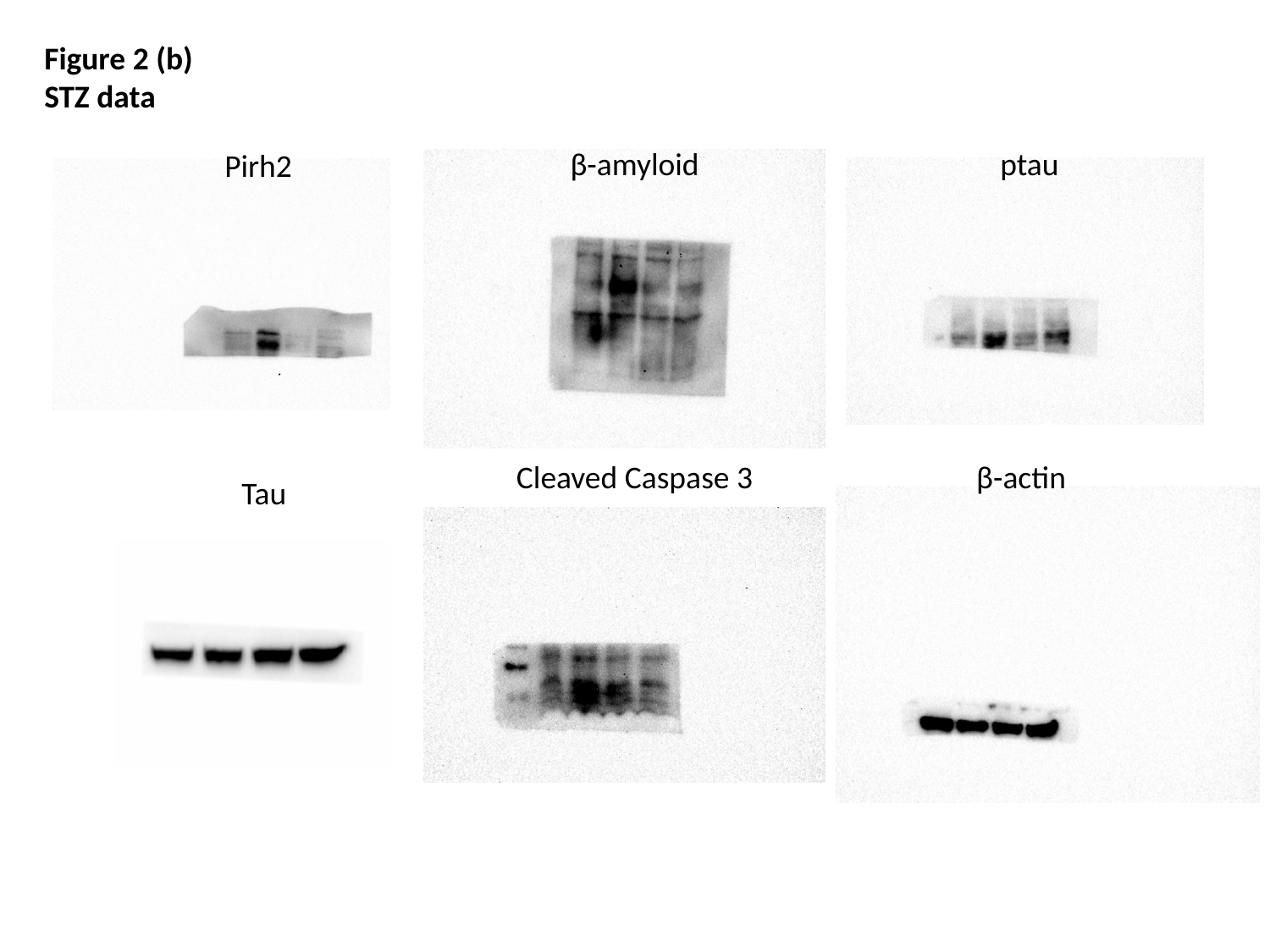

Figure 2 (b)
STZ data
β-amyloid
ptau
Pirh2
Cleaved Caspase 3
β-actin
Tau

## Slide 6
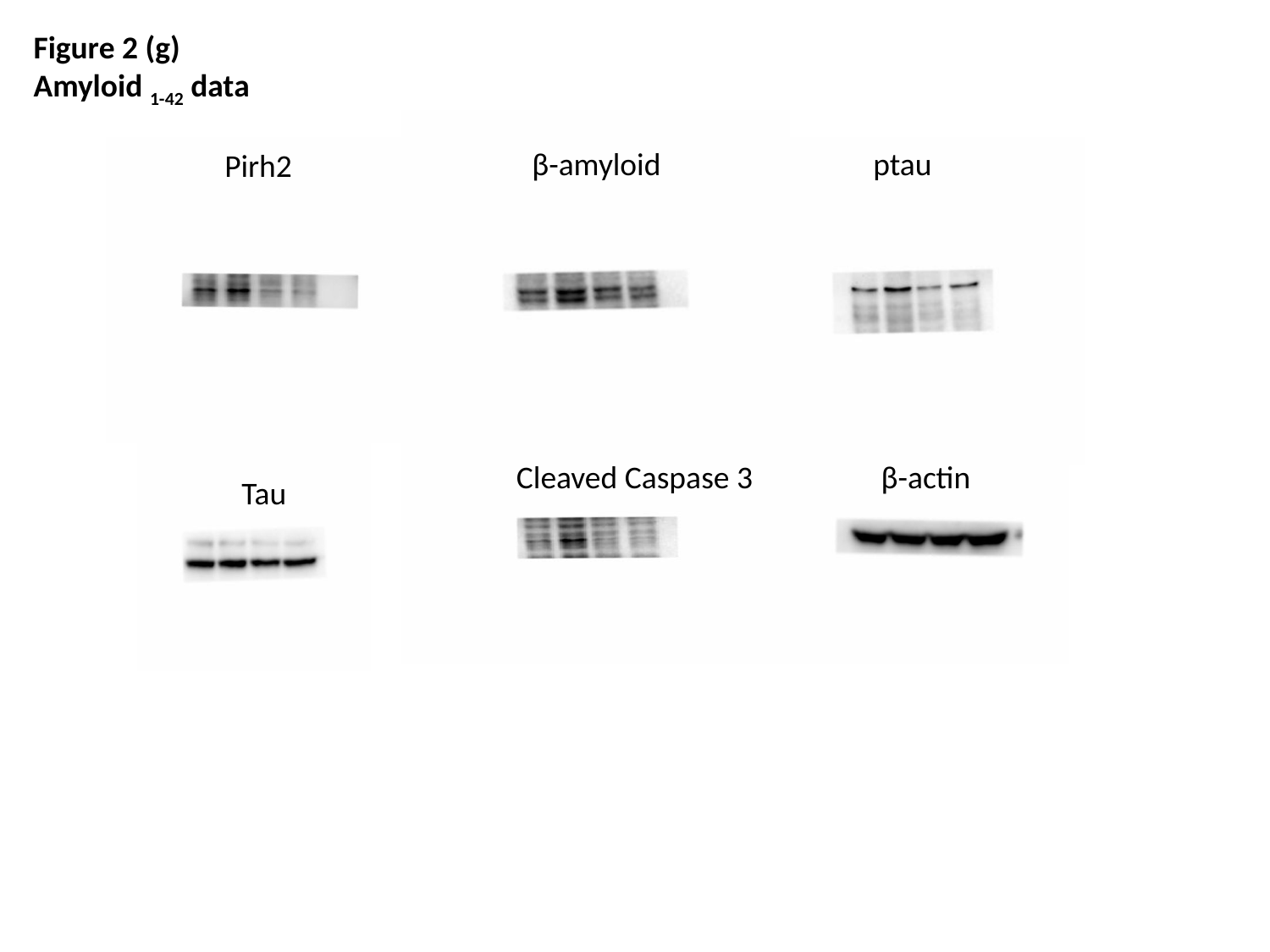

Figure 2 (g)
Amyloid 1-42 data
β-amyloid
ptau
Pirh2
Cleaved Caspase 3
β-actin
Tau

## Slide 7
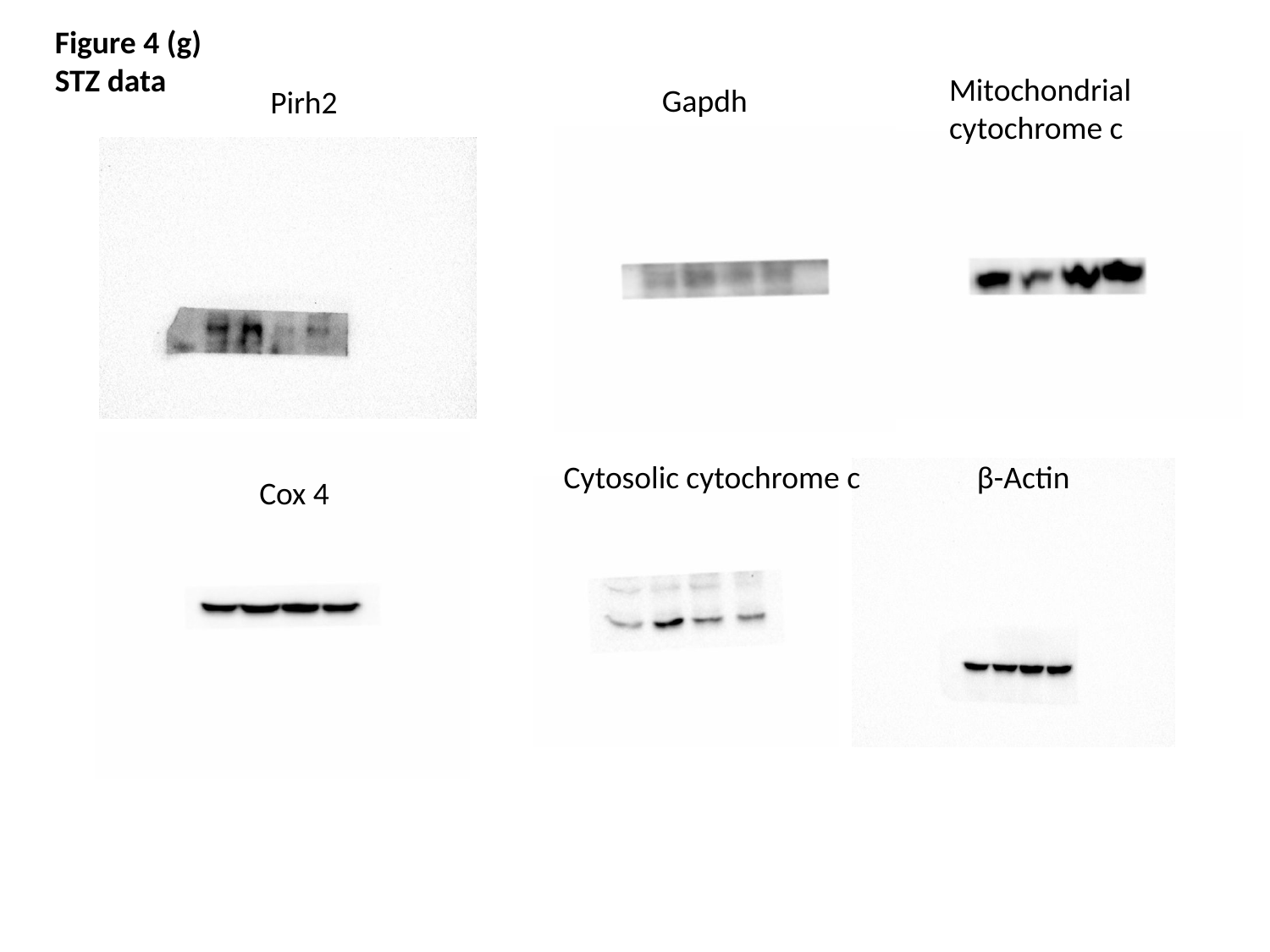

Figure 4 (g)
STZ data
Mitochondrial
cytochrome c
Gapdh
Pirh2
Cytosolic cytochrome c
β-Actin
Cox 4

## Slide 8
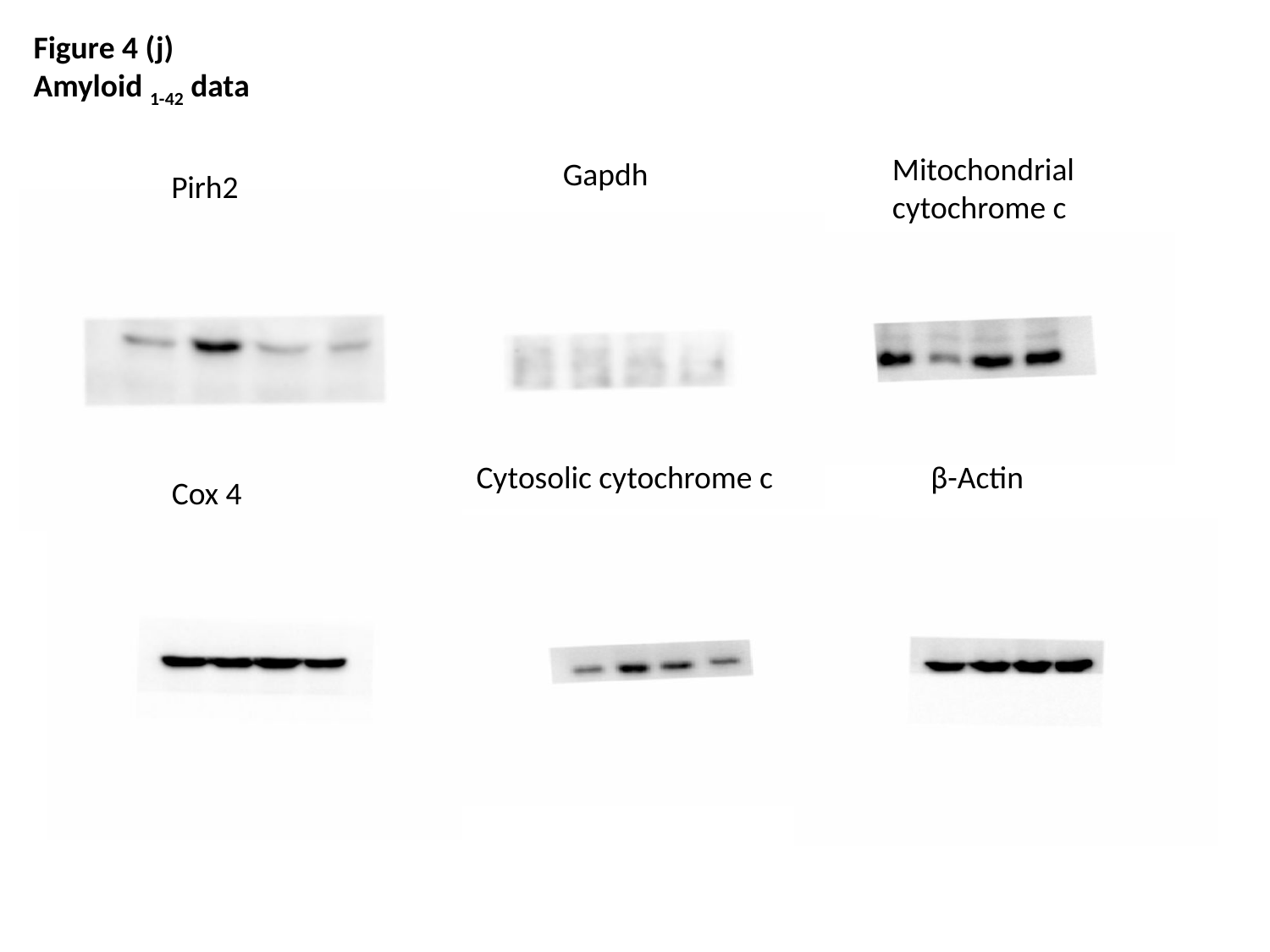

Figure 4 (j)
Amyloid 1-42 data
Mitochondrial
cytochrome c
Gapdh
Pirh2
Cytosolic cytochrome c
β-Actin
Cox 4

## Slide 9
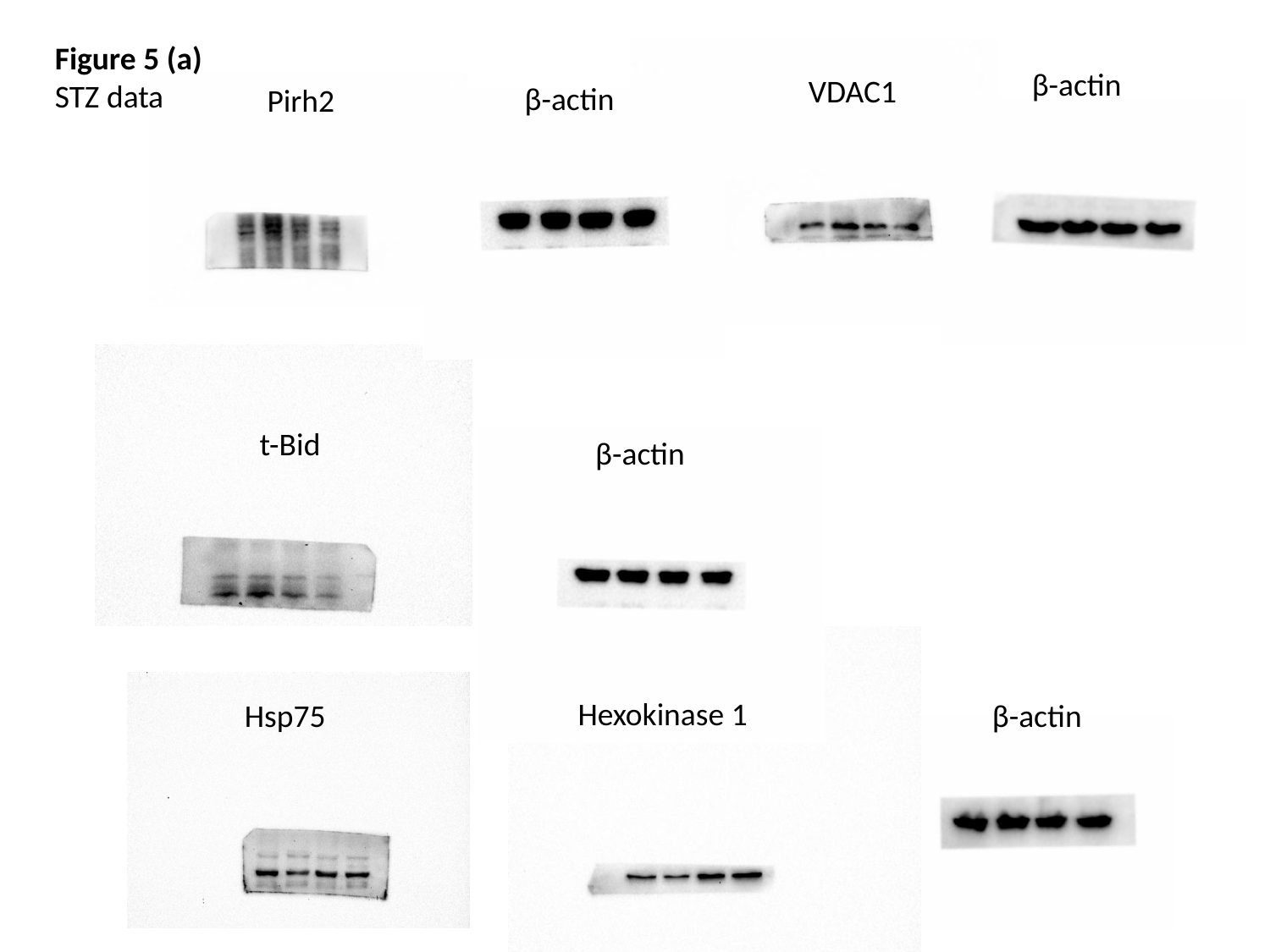

Figure 5 (a)
STZ data
VDAC1
β-actin
β-actin
Pirh2
t-Bid
β-actin
Hexokinase 1
Hexokinase 1
 Hsp75
β-actin

## Slide 10
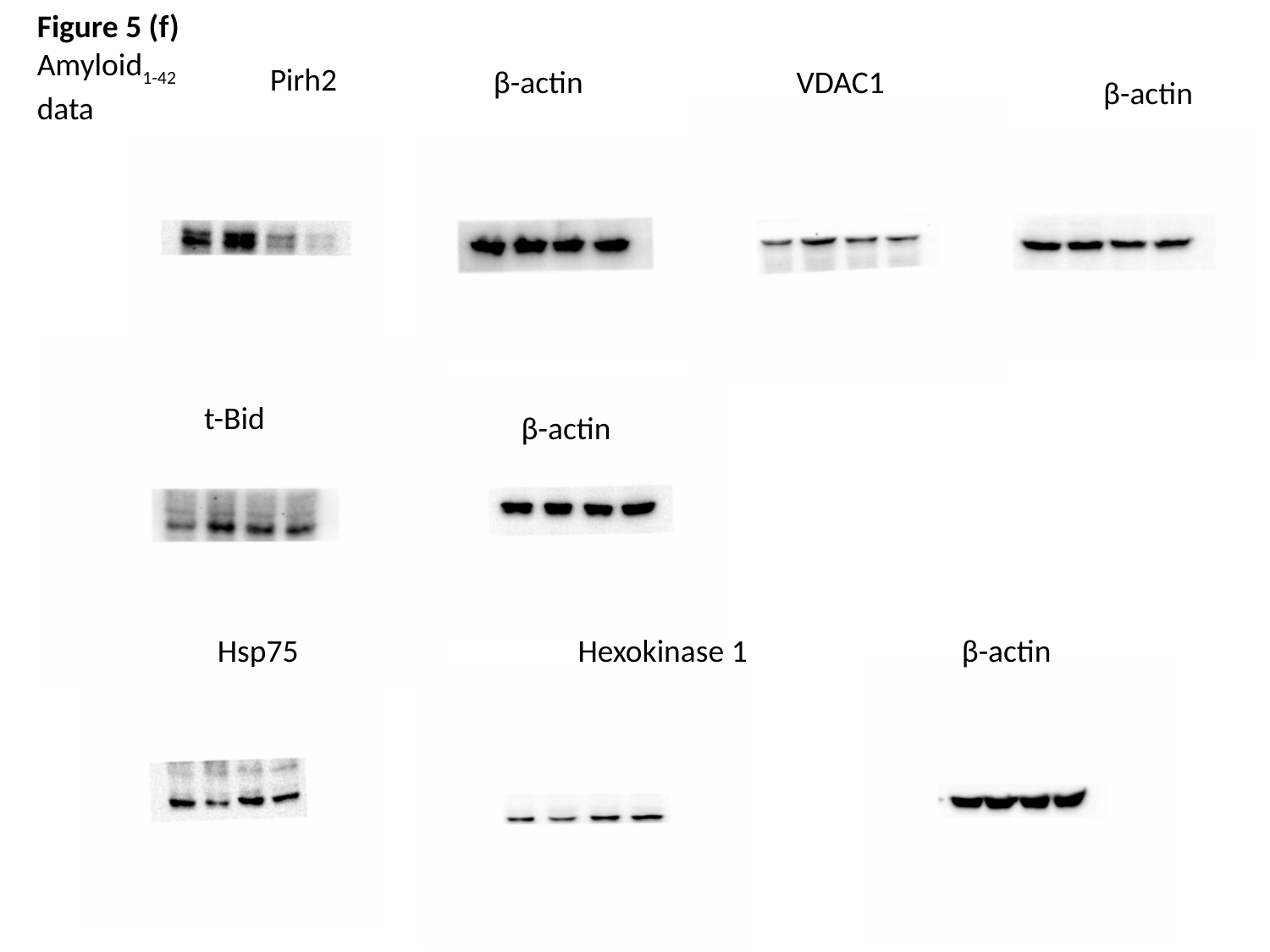

Figure 5 (f)
Amyloid1-42 data
Pirh2
β-actin
VDAC1
β-actin
t-Bid
β-actin
 Hsp75
Hexokinase 1
β-actin

## Slide 11
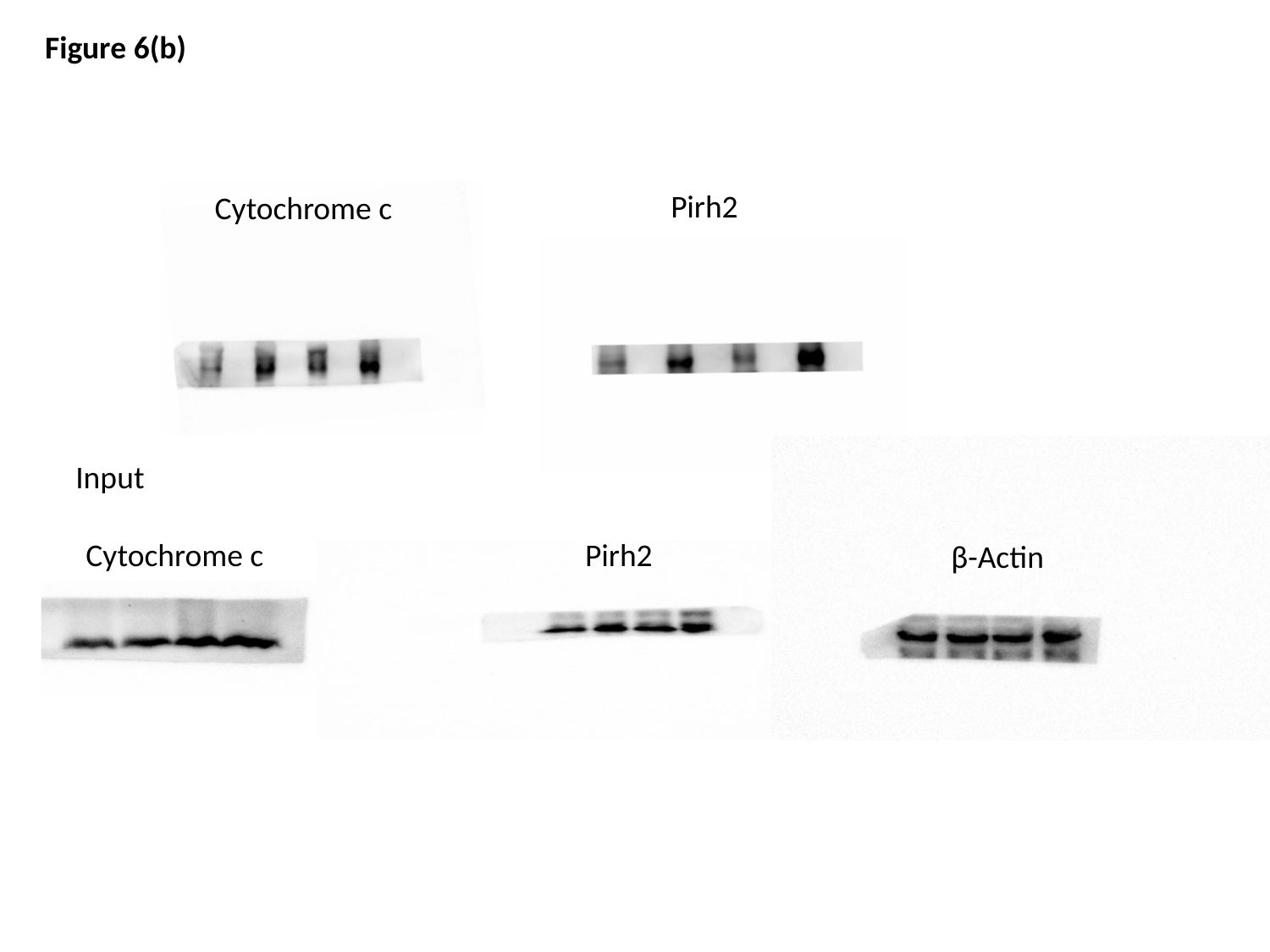

Figure 6(b)
Pirh2
Cytochrome c
Input
Cytochrome c
Pirh2
β-Actin

## Slide 12
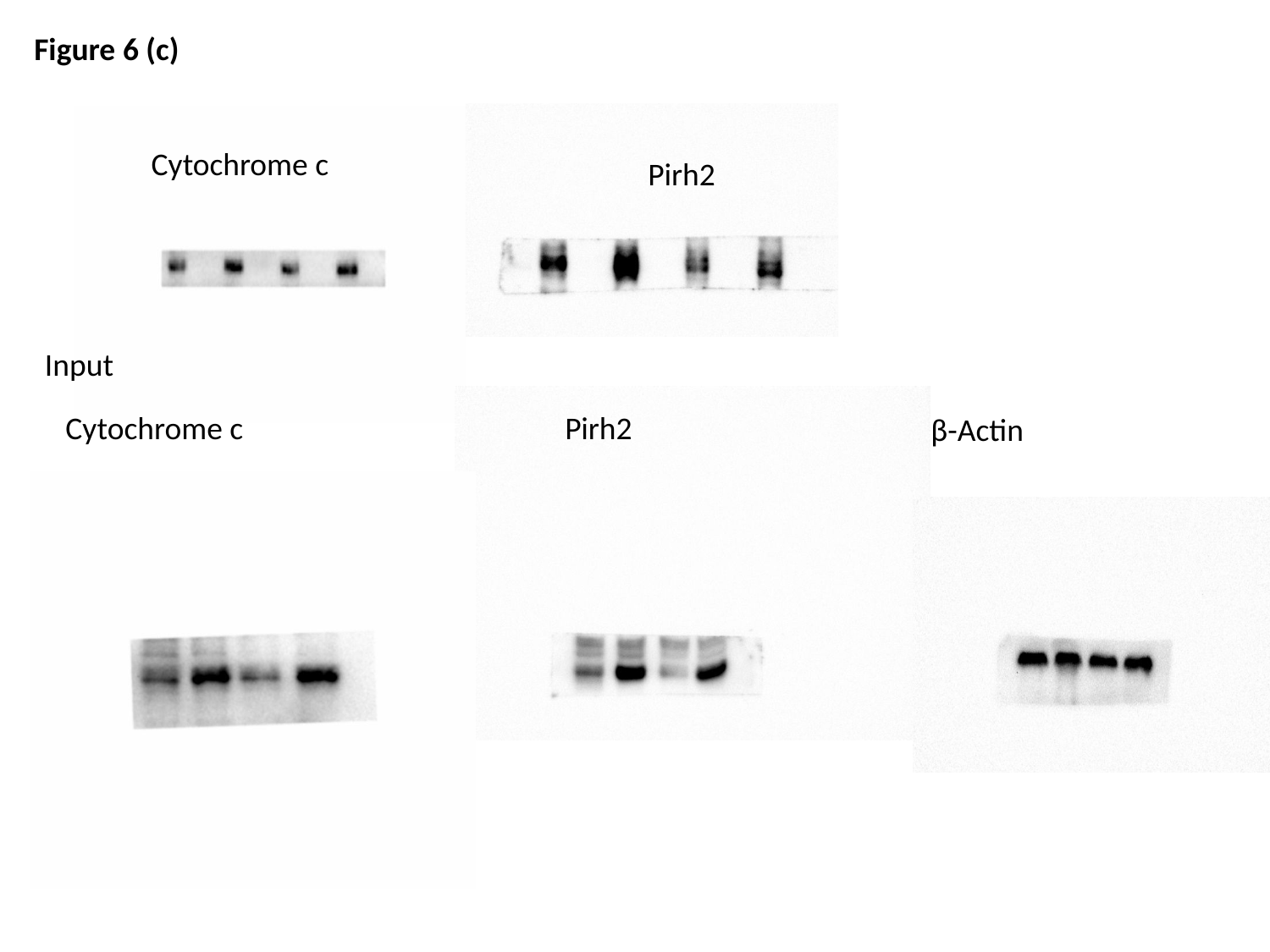

Figure 6 (c)
Cytochrome c
Pirh2
Input
Cytochrome c
Pirh2
β-Actin

## Slide 13
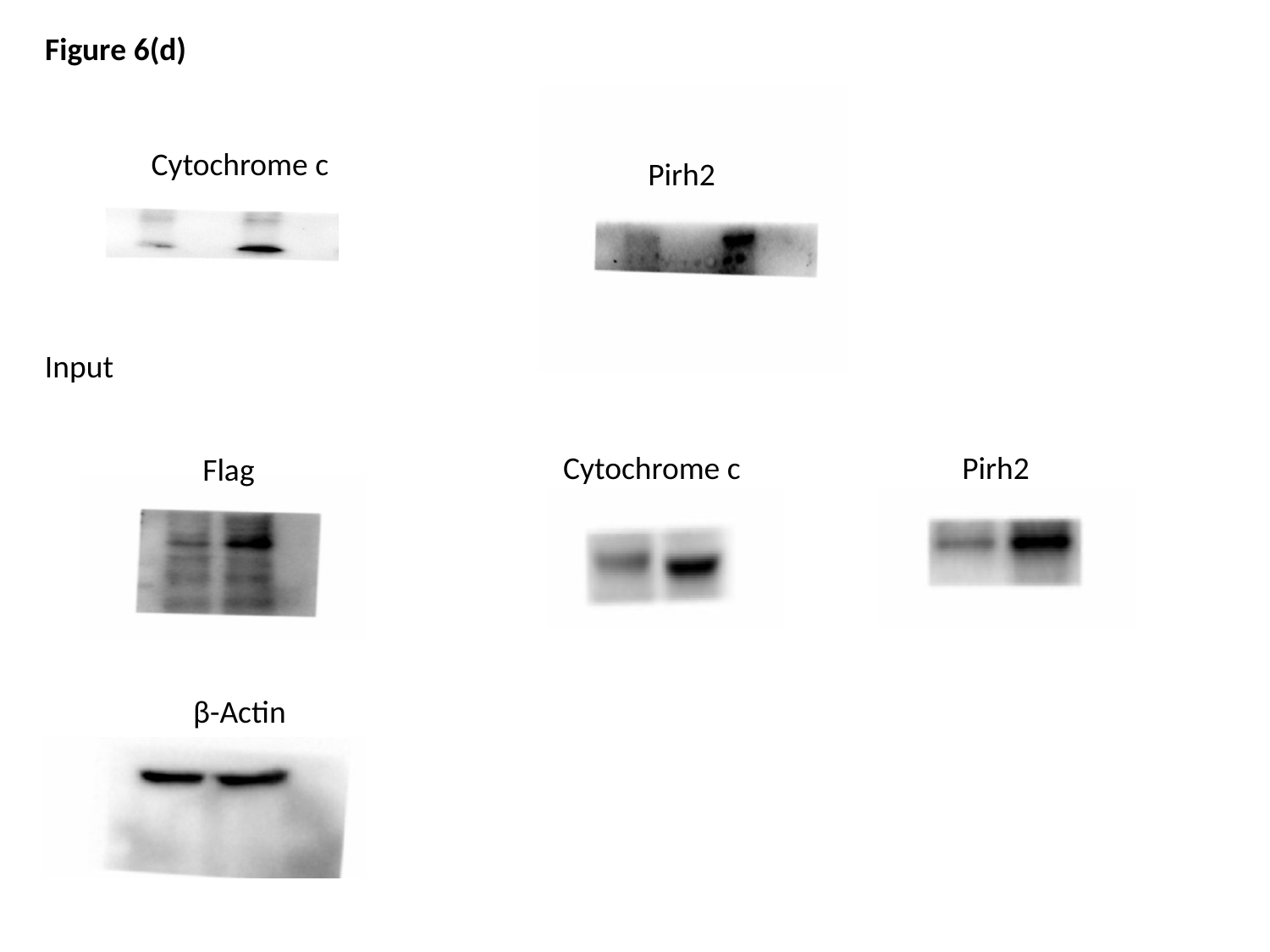

Figure 6(d)
Cytochrome c
Pirh2
Input
Cytochrome c
 Pirh2
 Flag
β-Actin

## Slide 14
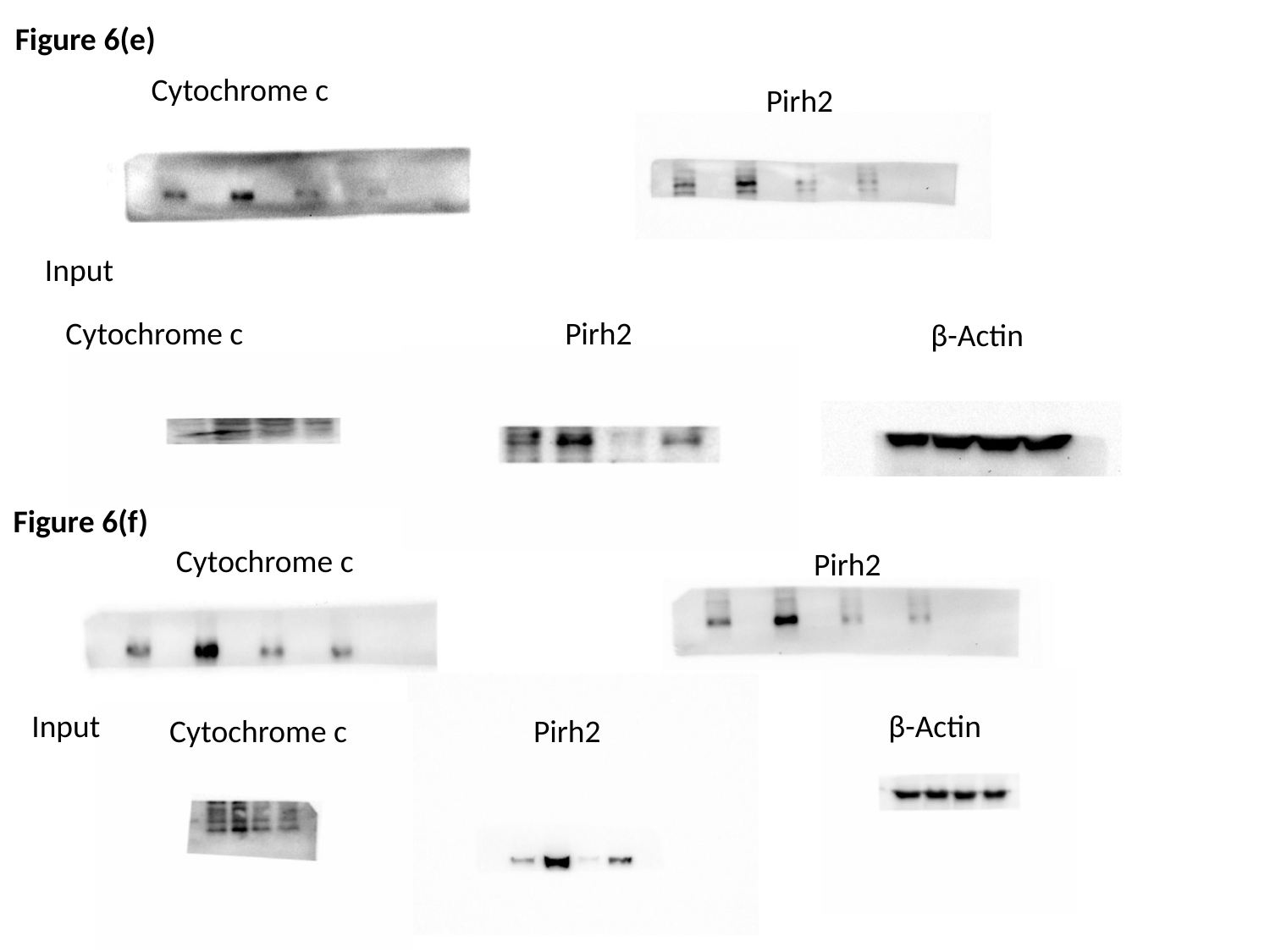

Figure 6(e)
Cytochrome c
Pirh2
Input
Cytochrome c
Pirh2
β-Actin
Figure 6(f)
Cytochrome c
Pirh2
Input
β-Actin
Cytochrome c
Pirh2

## Slide 15
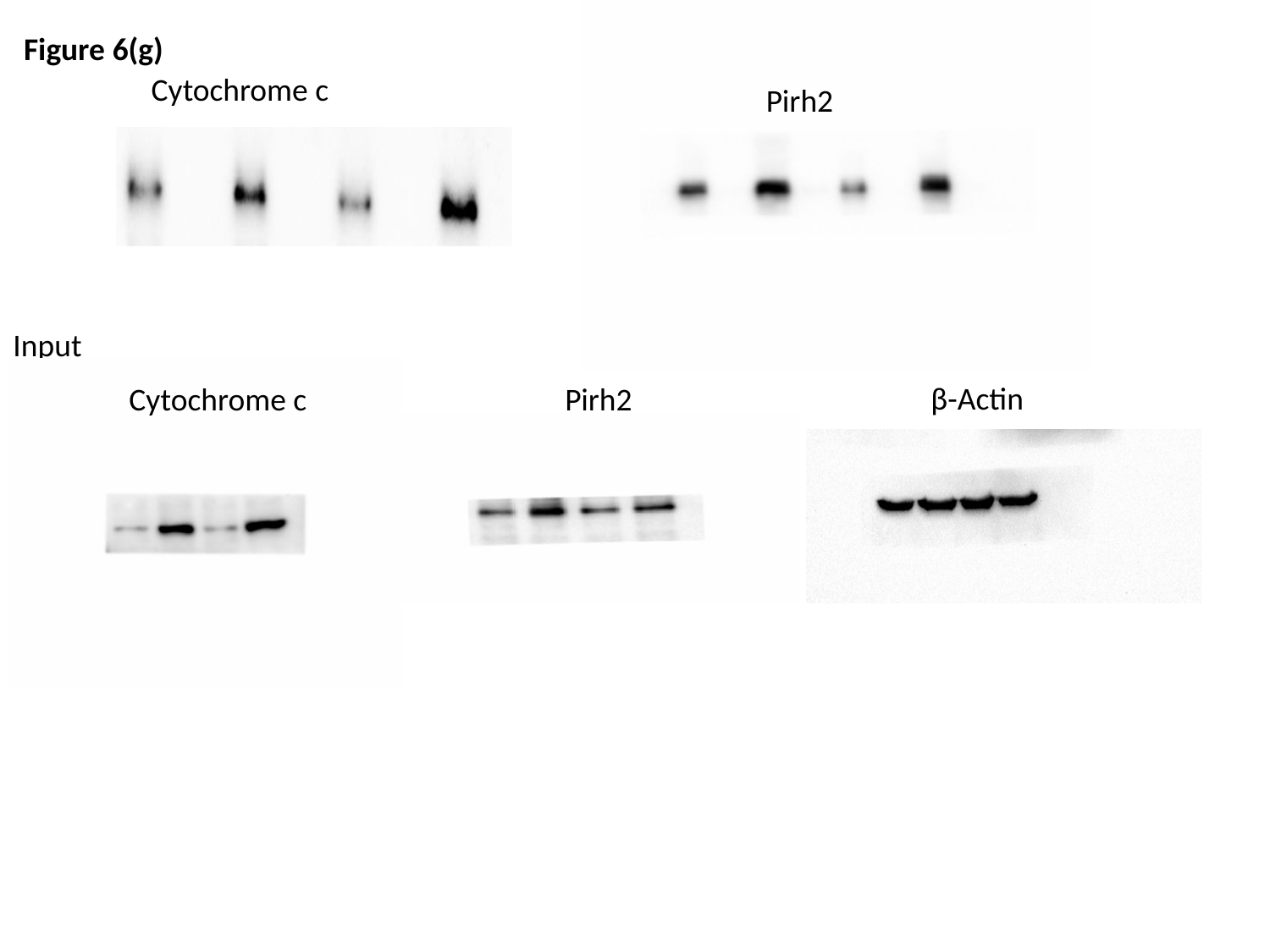

Figure 6(g)
Cytochrome c
Pirh2
Input
β-Actin
Cytochrome c
Pirh2

## Slide 16
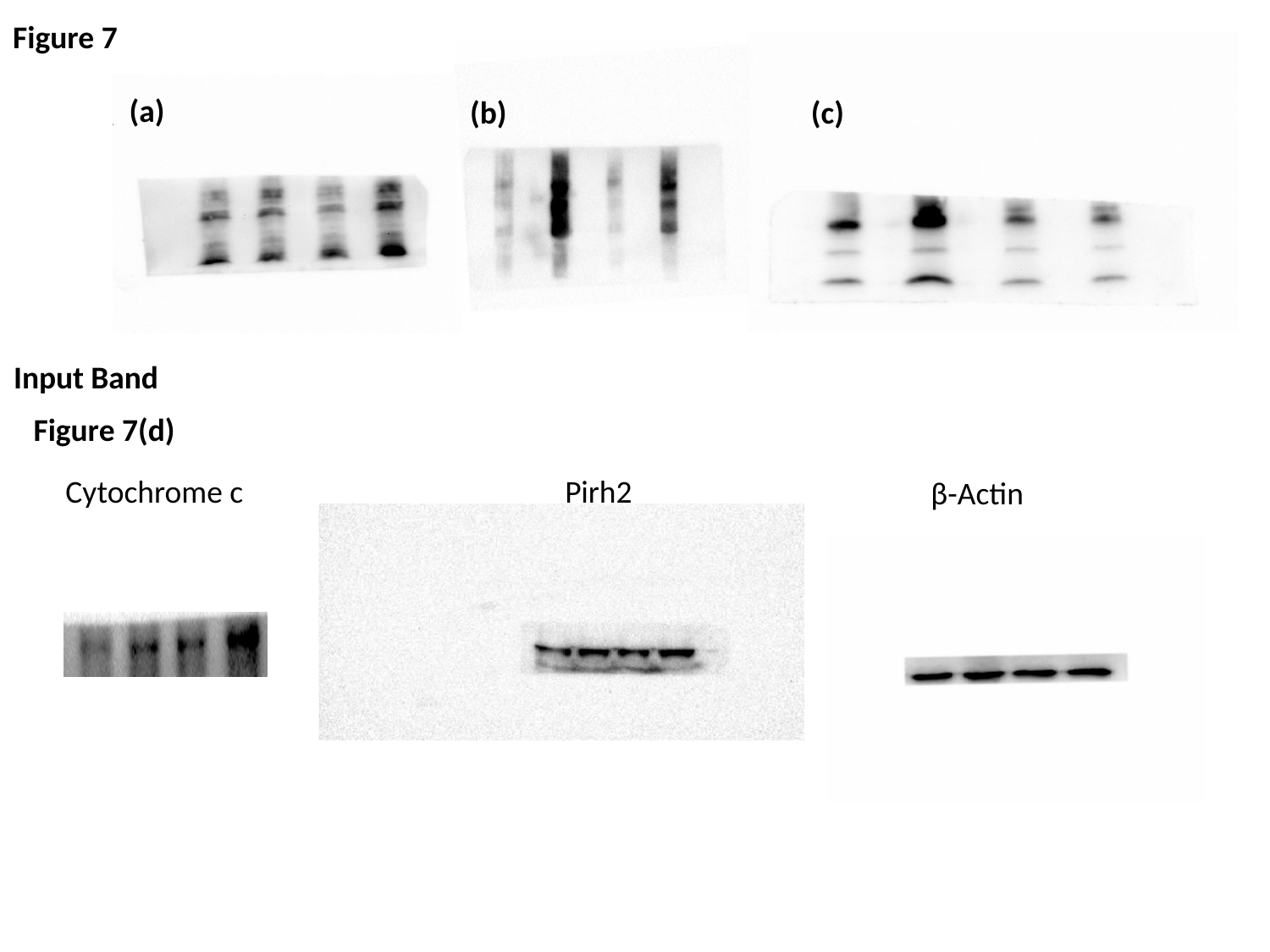

Figure 7
(a)
(b)
(c)
Input Band
Figure 7(d)
Cytochrome c
Pirh2
β-Actin

## Slide 17
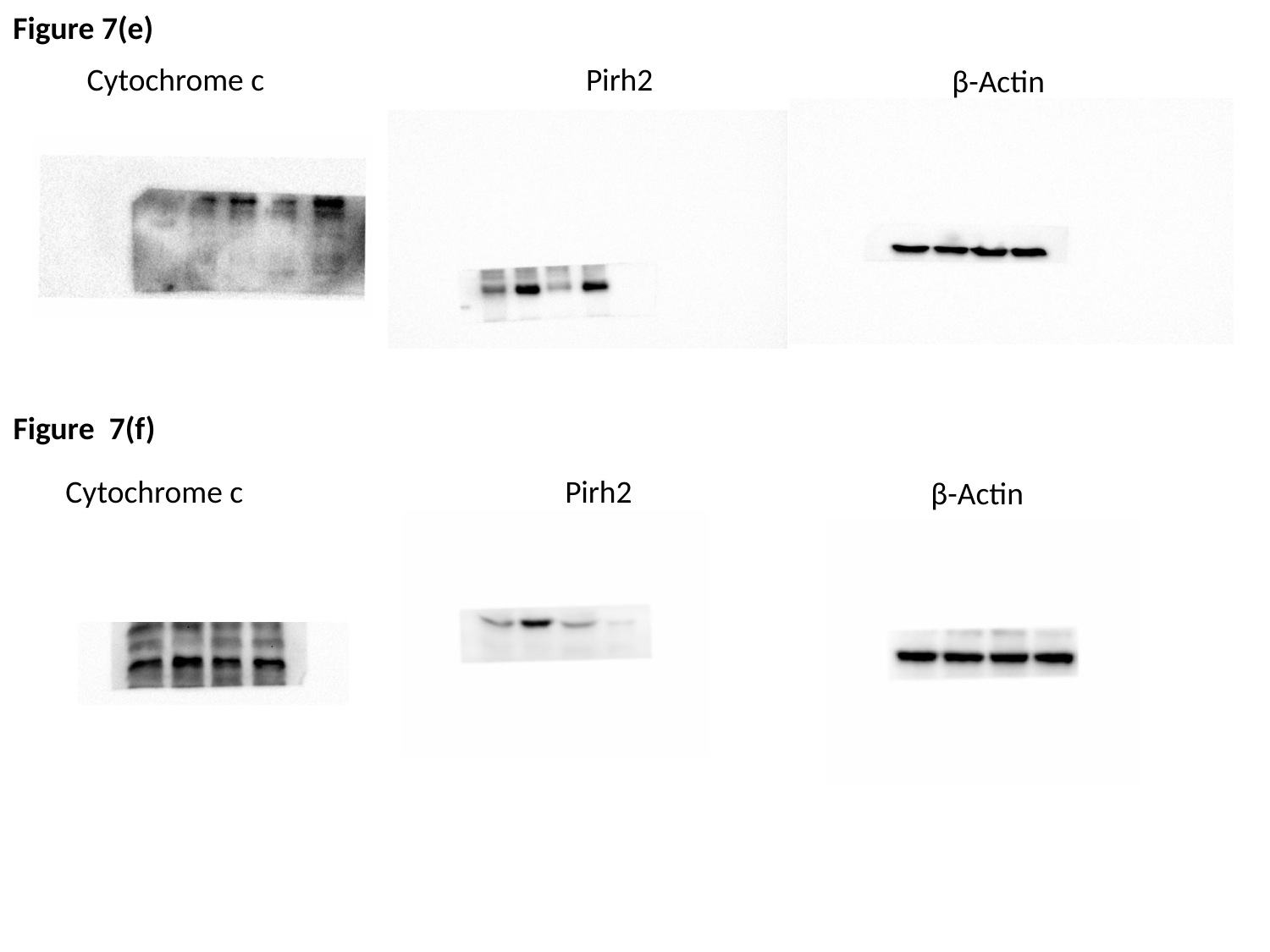

Figure 7(e)
Cytochrome c
Pirh2
β-Actin
Figure 7(f)
Cytochrome c
Pirh2
β-Actin

## Slide 18
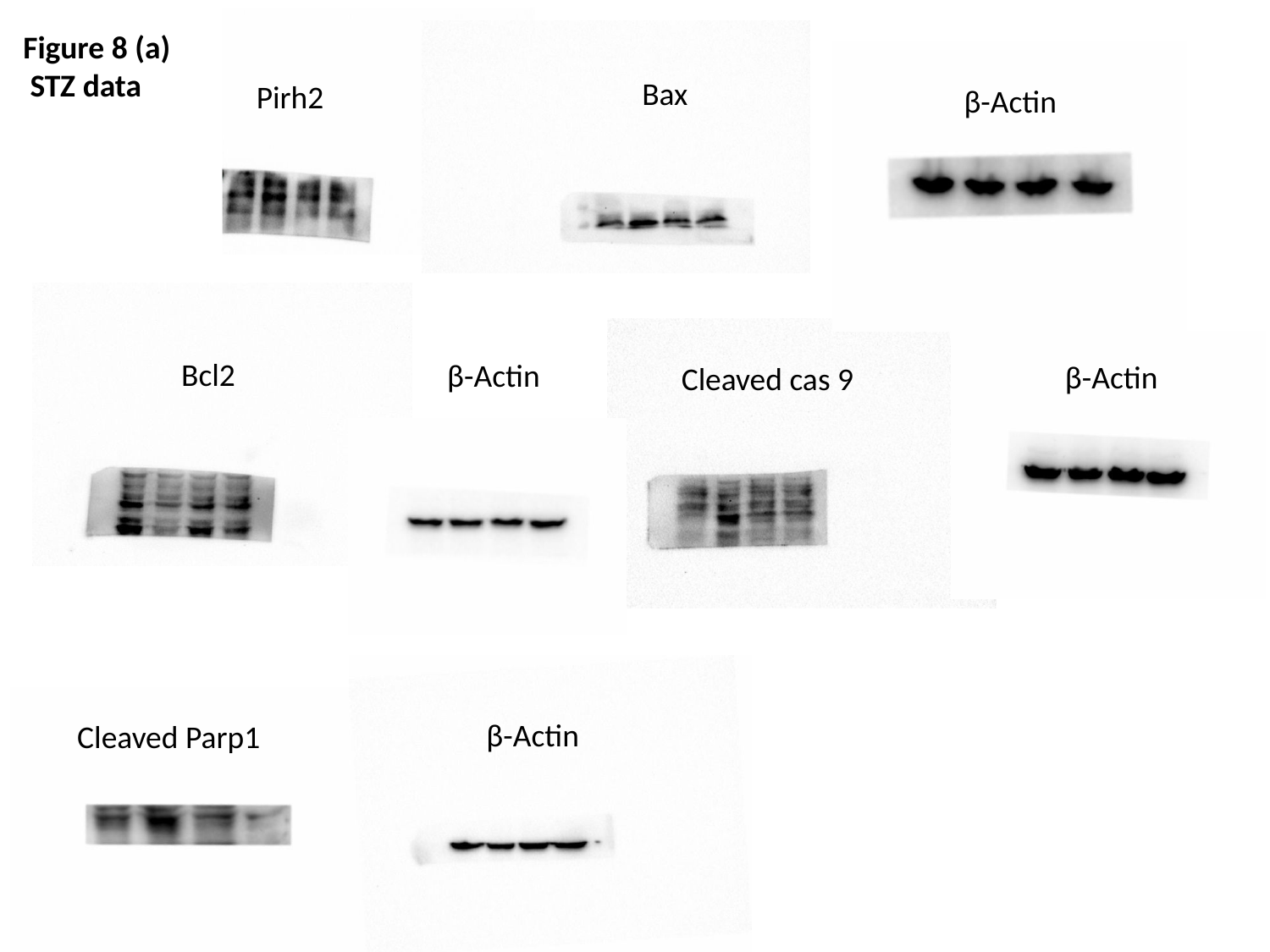

Figure 8 (a)
 STZ data
Bax
Pirh2
β-Actin
Bcl2
β-Actin
β-Actin
Cleaved cas 9
β-Actin
Cleaved Parp1

## Slide 19
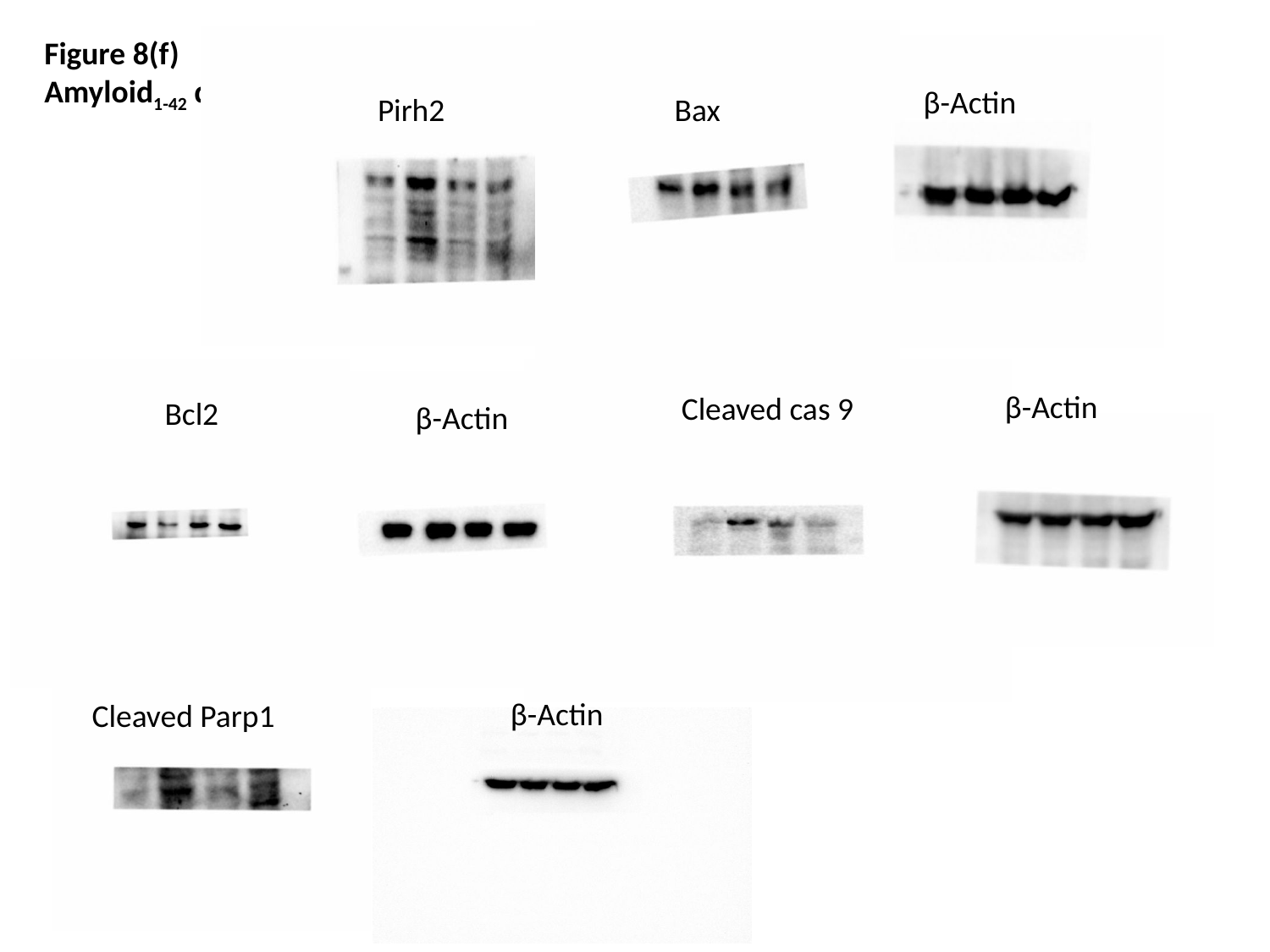

Figure 8(f)
Amyloid1-42 data
β-Actin
Pirh2
Bax
β-Actin
Cleaved cas 9
Bcl2
β-Actin
β-Actin
Cleaved Parp1

## Slide 20
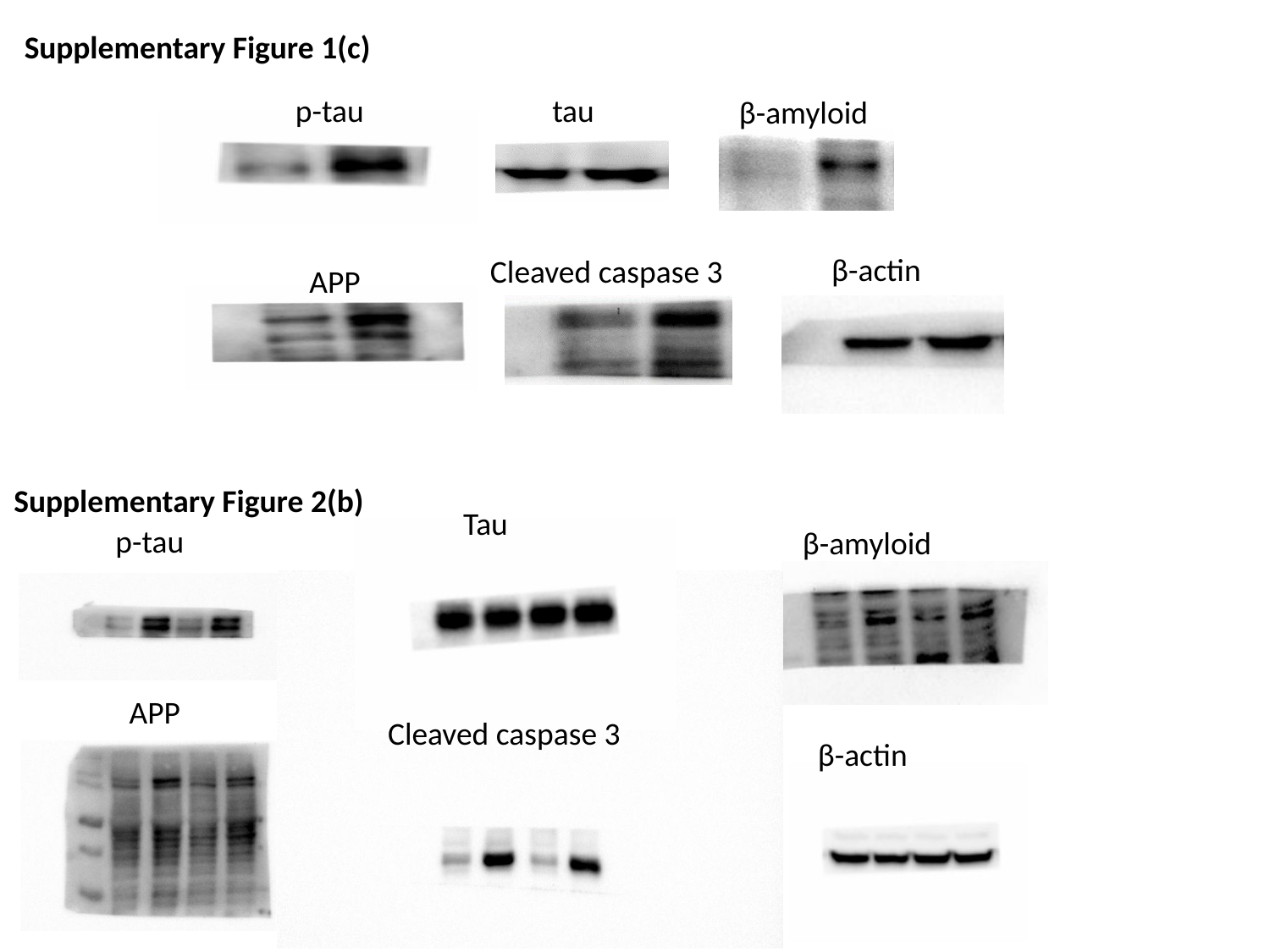

Supplementary Figure 1(c)
 p-tau
 tau
β-amyloid
β-actin
Cleaved caspase 3
APP
Supplementary Figure 2(b)
 Tau
 p-tau
β-amyloid
APP
Cleaved caspase 3
β-actin

## Slide 21
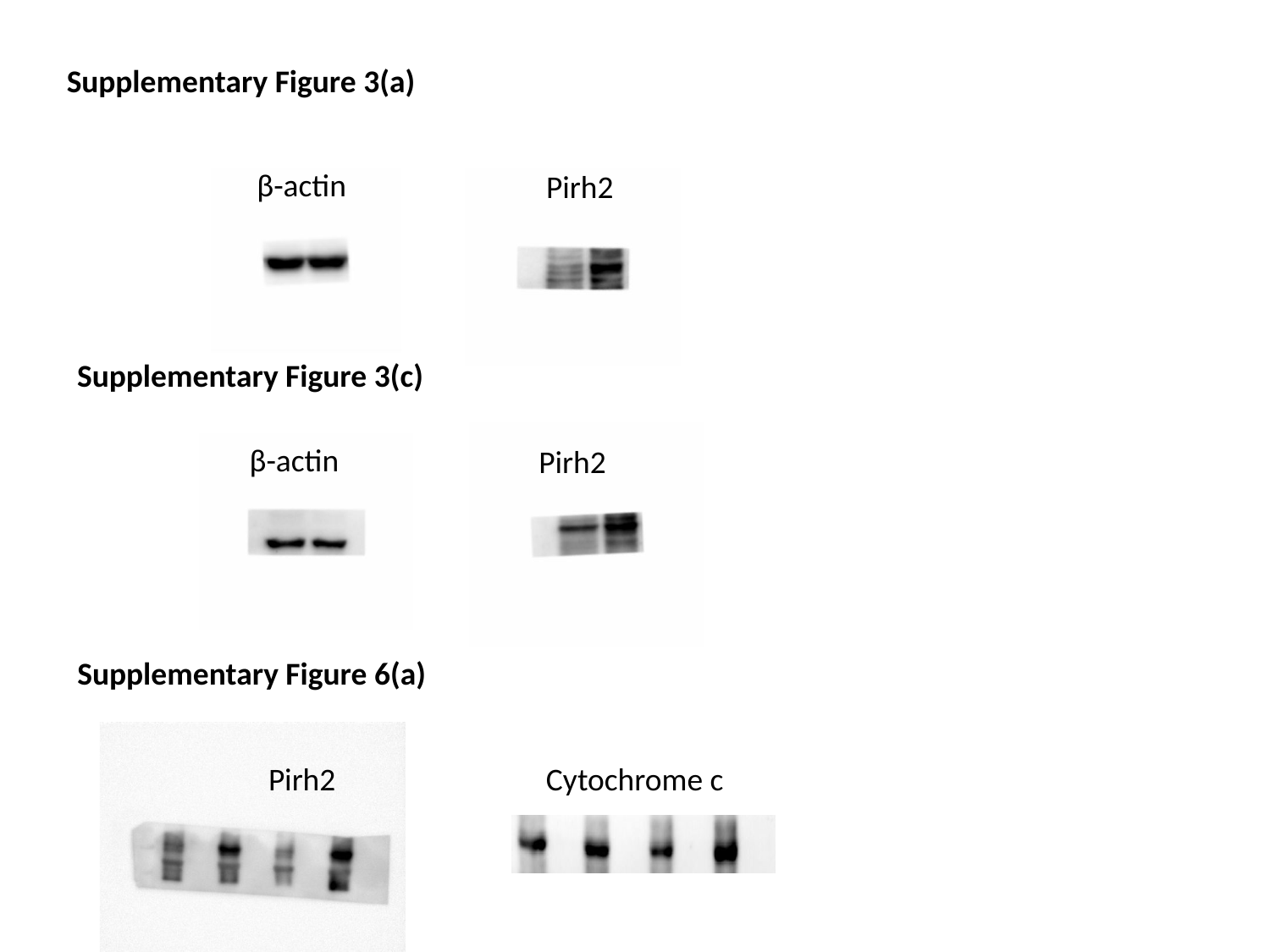

Supplementary Figure 3(a)
β-actin
 Pirh2
Supplementary Figure 3(c)
β-actin
 Pirh2
Supplementary Figure 6(a)
Pirh2
Cytochrome c

## Slide 22
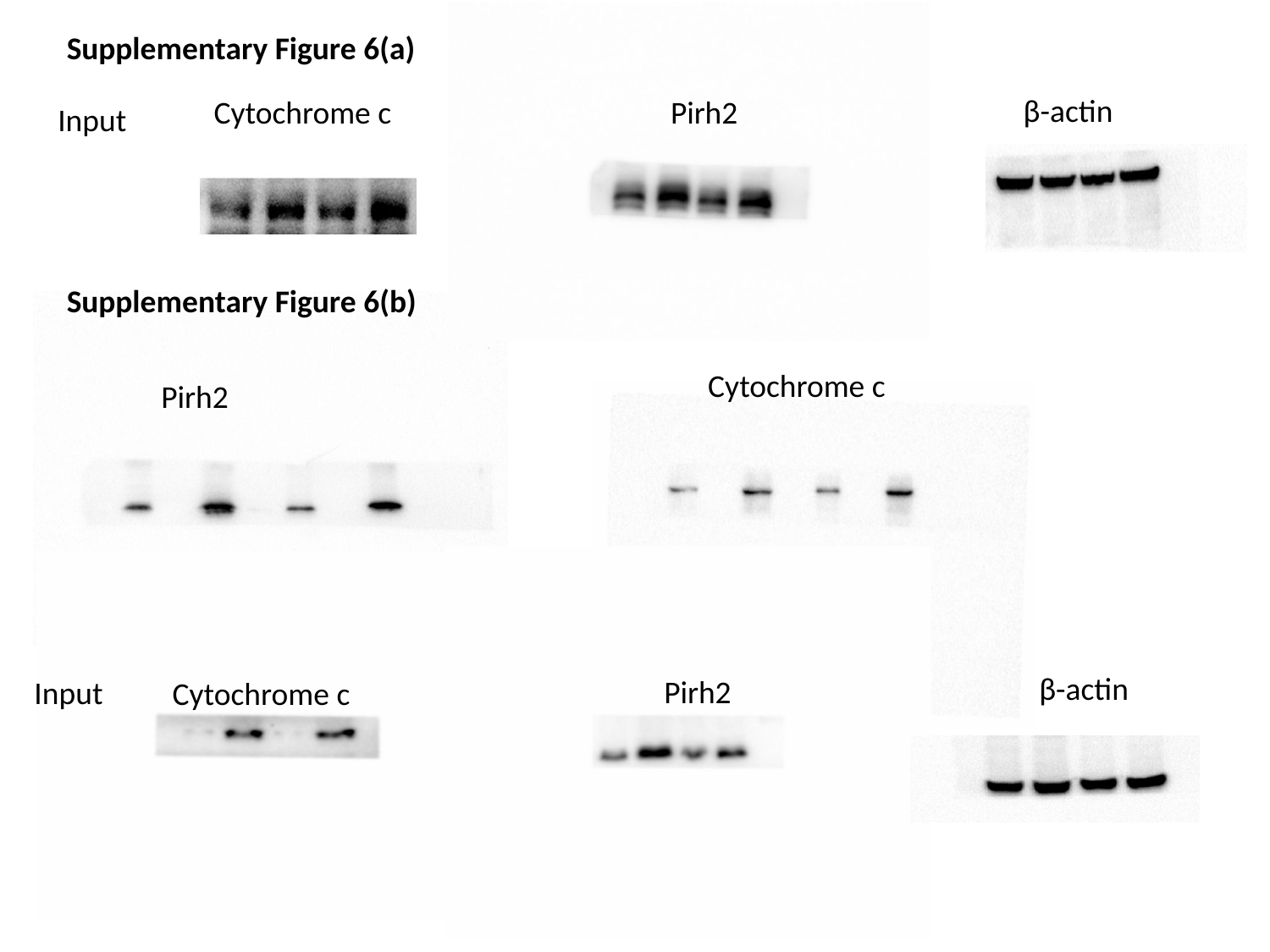

Supplementary Figure 6(a)
β-actin
Cytochrome c
 Pirh2
Input
Supplementary Figure 6(b)
Cytochrome c
Pirh2
β-actin
 Pirh2
Input
Cytochrome c
